# Potent inhibition of HIV replication in primary human cells by novel synthetic polyketides inspired by Aureothin

**DOI:** 10.1038/s41598-020-57843-9

**Published:** 2020-01-28

**Authors:** Alexander Herrmann, Manfred Roesner, Thomas Werner, Stefanie M. Hauck, Alisha Koch, Amelie Bauer, Martha Schneider, Ruth Brack-Werner

**Affiliations:** 1 Institute of Virology, Helmholtz Zentrum München - Deutsches Forschungszentrum für Gesundheit und Umwelt GmbH, Neuherberg, Germany; 2 mroe-consulting, Eppstein, Germany; 3 Department of Computational Medicine and Bioinformatics & Department of Internal Medicine, University of Michigan, Ann Arbor, Michigan USA; 4 Research Unit Protein Science, Helmholtz Zentrum München – Deutsches Forschungszentrum für Gesundheit und Umwelt GmbH, Munich, Germany

**Keywords:** HIV infections, Phenotypic screening

## Abstract

Overcoming the global health threat of HIV infection requires continuous pipelines of novel drug candidates. We identified the γ-pyrone polyketides Aureothin/Neoaureothin as potent hits by anti-HIV screening of an extensive natural compound collection. Total synthesis of a structurally diverse group of Aureothin-derivatives successfully identified a lead compound (**#7**) superior to Aureothin that combines strong anti-HIV activity (IC_90_<45 nM), photostability and improved cell safety. Compound **#7** inhibited *de novo* virus production from integrated proviruses by blocking the accumulation of HIV RNAs that encode the structural components of virions and include viral genomic RNAs. Thus, the mode-of-action displayed by compound **#7** is different from those of all current clinical drugs. Proteomic analysis indicated that compound **#7** does not affect global protein expression in primary blood cells and may modulate cellular pathways linked to HIV infection. Compound **#7** inhibited multiple HIV genotypes, including HIV-type 1 and 2 and synergistically inhibited HIV in combination with clinical reverse transcriptase and integrase inhibitors. We conclude that compound **#7** represents a promising new class of HIV inhibitors that will facilitate the identification of new virus-host interactions exploitable for antiviral attack and holds promise for further drug development.

## Introduction

With over 37 million individuals globally living with HIV, HIV infection represents a serious public health threat. HIV treatment was revolutionized by the development of potent antiviral drugs. Their availability and use in combination antiretroviral therapy (cART) has turned a positive HIV diagnosis from a certain death sentence to a manageable disease^1^. Successful drug therapy (i.e. sustained suppression of virus load to undetectable levels) has also been shown to dramatically limit rates of transmission in serodiscordant couples^2,3^. Furthermore, antiviral drugs taken as prophylactic measure prior to exposure strongly reduce the risk of virus transmission^1^, further demonstrating the importance of anti-HIV drugs for transmission control. Overall, anti-HIV drugs are key to controlling and overcoming the HIV pandemic and preventing AIDS.

The main goal of cART is to suppress virus replication to undetectable levels and to minimize the emergence and transmission of drug resistant variants. Unfortunately, global HIV drug resistance is on the rise^4^. This pertains both to pre-treatment drug resistance levels, which in 6 of 11 countries exceeded 10%, as well as prevalence of drug resistance acquired during cART treatment (4–28%). Therefore, a major target of the global action plan for overcoming HIV/AIDS is the intensification of research for the development of new treatments and drugs. These are also needed to improve individualization of antiretroviral therapy and to block HIV production by cell populations with persisting, integrated HIV proviruses.

Clinical anti-HIV drugs interfere with the HIV replication cycle at the steps of HIV entry, reverse transcription, integration and maturation of virus particles after release from the host cell. Most clinical drugs inhibit steps of the replication cycle catalysed by HIV enzymes (i.e. protease, integrase and reverse transcriptase). cART combines three active drugs, of which two are nucleotide or nucleoside reverse transcriptase (NRTI) inhibitors and the third is from a different class, i.e. a non-nucleoside reverse transcriptase inhibitor (NNRTI), protease or integrase inhibitor^1^. Currently no approved clinical drugs prevent expression of virion components from integrated proviruses.

Natural products provide a rich source of novel structures for drug discovery^5^. About 50% of drugs approved by the FDA between 1981 and 2010 were derived from or inspired by natural compounds. These also include a number of anti-infective drugs.

The aim of this study was to identify new anti-HIV structures that differ both chemically and functionally from current therapeutic drugs. We report a new class of synthetic HIV inhibitors with a scaffold based on the natural polyketide Aureothin. HIV inhibitory activity of prototypical compound **#7** was apparent in primary HIV-target cells, directed against multiple HIV genotypes, including clinical HIV-type 1 and HIV-type 2 isolates, and synergized with clinical anti-HIV drugs. Compound **#7** treatment inhibited expression of HIV structural components on protein and RNA levels. We conclude that this class of compounds has the potential to advance the development of novel anti-HIV therapeutic agents and to expand options for anti-HIV treatment.

## Results

### Identification of bacterial γ-pyrone polyketides (Aureothin/Neoaureothin) as potent HIV inhibitors

To identify natural compounds with anti-HIV activity we screened a collection of 10,000 natural compounds: Anti-HIV activity was evaluated by determining compound effects on infectious virus production by LC5-RIC cells which are highly susceptible for HIV and contain a DsRed1-encoding reporter gene activated by HIV-infection^6–8^. For screening, cultures of LC5-RIC reporter cells were first exposed to virus in the presence of the compound (first-step of the assay). Subsequently, supernatants of these cultures were transferred to secondary plates with uninfected LC5-RIC cells. The fluorescent signal of the cultures in the secondary plates served as a measure of compound effects on infectious virus production.

The polyketide Aureothin and the related molecule Neoaureothin (Spectinabilin) were identified as hit candidates by single-dose screening (10 µM) and validated by assessment of dose-dependency of HIV-inhibitory activities. Aureothin (IC_50_ = 5.3 ± 0.40 nM) and Neoaureothin (IC_50_ = 2.2 ± 0.06 nM) showed very potent anti-HIV activity, which was comparable or only moderately lower than that of several clinical drugs assayed in parallel (Protease inhibitor Saquinavir^9^, IC_50_ ~ 10 nM); reverse transcriptase inhibitor Efavirenz^10^ and integrase inhibitor Dolutegravir;^11^, each ~ 1 nM)

Based on the interesting anti-HIV activities of these hit candidates, we decided to explore the anti-HIV potential of Aureothin and related polyketides in more detail.

### Design and activity profiling of synthetic aureothin derivatives

The natural product Aureothin, which is produced by *Streptomyces thioluteus*^12^, harbours a γ-pyrone group and a tetrahydrofuran ring separated from a 4-nitro phenyl group by a diene backbone (Table 1, **#1**). The nitro group in the aryl moiety bears high risk for toxicity^13^ which is a liability for drug development. We decided to investigate structure-activity-relationships (SAR) of a broader scope of new, more drug-like Aureothin analogues.

**Table 1:** Inhibitory activities of synthetic Aureothin-derivatives against HIV infection of human primary blood-derived mononuclear cells (PBMCs). Shown are the molecular structures of all Aureothin-derivatives, their half maximal (IC_50_) and 90% (IC_90_) HIV-inhibitory concentrations,  resp. R is depicted in the structure of Aureothin (#1). Compound effects were evaluated in human PBMCs exposed to HIV-1_LAI_. Shown are the means and standard deviations (±SD) of at least three independent experiments with triplicates (n ≥ 3; m =3). Each experiment was performed with PBMCs from a different donor.

#	Mol. Structure	IC_50_ (nM)	IC_90_ (nM)	#	Mol. Structure	IC_50_ (nM)	IC_90_ (nM)	#	Mol. Structure	IC_50_ (nM)	IC_90_ (nM)
1		11.7 ± 1.37	45.0 ± 3.33	6		14.3 ± 1.36	86.0 ± 3.06	14		22.4 ± 1.10	74.1 ± 2.42
				7		10.3 ± 0.65	41.0 ± 1.23	15		40.1 ± 3.76	242.2 ± 3.20
				8		48.7 ± 9.65	587.6 ± 148.20	16		74.9 ± 10.92	269.8 ± 3.84
				9		1,261.0 ± 107.80	3,939 ± 3.66	17		4,080 ± 1,073	141,326 ± 3,378
2		> 10,000	—	10		959.3 ± 222.6	8,411 ± 5.34	18		10,754 ± 2,231	240,907 ± 2,155
3		0.5 ± 0.19	2,937 ± 983.30	11		> 10,000	—	19		53.9 ± 3.51	463.5 ± 2.80
4		4,021.0 ± 685.80	14,102 ± 6.74	12		1.5 ± 0.11	74.0 ± 0.87	20		41.7 ± 11.02	485.2 ± 4.14
5		561.0 ± 101.20	4,211 ± 4.41	13		3.6 ± 1.54	1,818 ± 3.29	21		14.6 ± 0.98	189.2 ± 0.82

Following the principle route of Jacobsen *et al*.^14,15^, we synthesized 20 derivatives, including electron-withdrawing and -donating, as well as lipophilic and polar substituents at ortho, meta and/or para position of the aryl moiety (Table 1, Supplementary data file S1). In short, appropriately substituted benzaldehydes were converted to the substituted 2,2-dibromovinylbenzenes, and were reacted with the boronic ester of the key intermediate γ-pyrone-furanone (compound **#2**)^14,15^, followed by Br-methyl conversion with Zn(CH_3_)_2_ and Pd-phosphin catalyst. Analysis of anti-HIV activities of these synthetic derivatives with LC5-RIC cells identified 6 compounds (**#3**, **#6, #7, #12**, **#13**, and **#15**) that retained half maximal (IC_50_) anti-HIV activities similar to Aureothin (IC_50_ below 10 nM) as well as other, less active derivatives (Supplementary Table S1). Profiling of anti-HIV activities of all Aureothin derivatives was then repeated using primary human target cells for HIV, i.e. peripheral blood mononuclear cells (PBMC), in at least three independent assays with PBMCs from different donors as described in Materials and Methods (*Inhibition of infection assays*). Compounds (**#3**, **#6, #7, #12**, **#13**, and **#21**) showed half maximal (IC_50_) anti-HIV activities at similar concentrations as Aureothin (**#1**) in PBMCs (Table 1). However, determination of concentrations required for 90% inhibition of HIV infection of PBMC revealed an IC_90_ value comparable to Aureothin only for compound **#7**.

The other compounds in this collection showed lower anti-HIV activities than Aureothin. As expected, a truncated compound lacking the nitro-aryl moiety and the linker (**#2**) showed no activity at concentrations up to 10 µM. Replacement of the nitro-group with a carboxyl group in a full-length compound also led to a dramatic loss of activity (**#18**), underlining the importance of the nature of the para-substituent in the aryl-moiety for inhibition of HIV infection.

**Figure 1:**
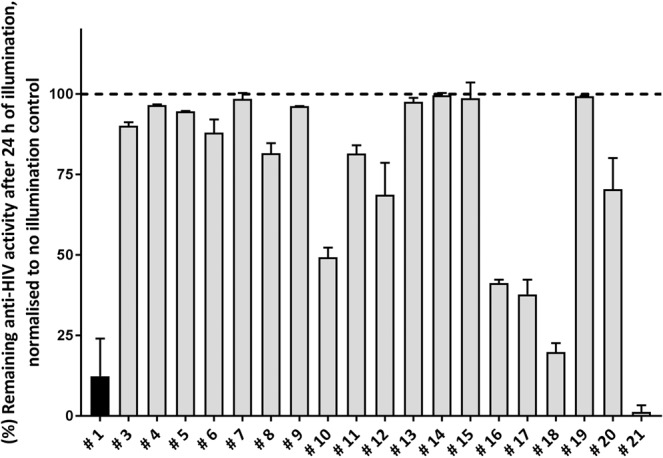
Profiling of photostability of anti-HIV activity of Aureothin and synthetic Aureothin-derivatives. Anti-HIV activity of compound samples illuminated for 24 h were normalised to anti-HIV activity of samples of the same compound kept in the dark. Anti-HIV activity was measured by quantifying the amounts of infectious virus produced by HIV-1_LAI_ exposed LC5-RIC cells treated with compounds at IC_90_ concentrations (Supplementary Table S1). The black column shows data for Aureothin (**#1**) and the grey columns for Aureothin-derivatives. Shown are means of at least three independent experiments with biological triplicates (n ≥ 3; m = 3) and the standard error of the mean (SEM) indicated.

Neoaureothin was previously reported to be photolabile, showing light-induced isomerization-cyclization^16^. Therefore, we investigated the photostability of Aureothin/Neoaureothin and derivatives by comparing their anti-HIV activities after exposure to light for 24 h with those of the same compounds kept in the dark. The high-activity compound **#7** retained over 95% anti-HIV activity after exposure to light, similar to several other synthetic compounds (**#4**, **#9**, **#13**, **#14**, **#15**, **#19**) (Fig. 1). In contrast, Aureothin (**#1**) and several synthetic compounds (**#10, #16, #17, #18**, and **#21**) showed ≥50% loss of anti-HIV-activity compared to the non-illuminated controls (Fig. 1). Photostable compounds included both compounds with high and low anti-HIV activity, indicating that the chemical determinants of photostability and anti-HIV activity are not identical.

Cell safety of Aureothin derivatives in HIV-exposed PBMC cultures was profiled in various assays commonly used to evaluate adverse off-target effects of compounds on cells^17^. Cytotoxic effects were apparent for the natural product Aureothin, using the CellTiter-Glo^®^ Viability Assay (Supplementary Fig. S1; Supplementary Table S2). The 50% cytotoxic concentration (CC_50_) was ~2.27 µM, indicating a selectivity index (CC_50_/IC_50_) of ~194 for Aureothin (**#1**). In contrast, none of the most potent synthetic Aureothin derivatives showed cytotoxic effects up to 10 µM. This resulted in a selectivity index>970 for compound **#7**, which is 5 times higher than for Aureothin.

These results indicate that replacing the nitro-group at the phenyl ring strongly improves cell safety of these molecules in PBMCs.

Taking together all improvements, the 4-trifluoromethyl substituted compound **#7** was selected for further in-depth characterization because of its excellent anti-HIV activity, high photostability and low cytotoxicity.

### Mode-of-action of compound #7 in the HIV replication cycle

The reporter gene in LC5-RIC cells contains the HIV promoter and other regulatory elements of HIV expression and is activated by early viral gene expression during HIV infection^6^. Use of LC5-RIC cells in a previously described two-step assay format allows comparison of the effect of compound treatment on expression of the DsRed1 reporter gene and infectious virus production in the same cultures. As reference compound, we used an experimental inhibitor of HIV transcription called Flavopiridol. Flavopiridol blocks pTEFb, an essential co-factor for Tat-dependent activation^18,19^. Experiments were performed with 50 nM of either compound, at which both compounds display potent anti-HIV activity and are nontoxic in LC5-RIC cells (see Supplementary Table S2).

**Figure 2:**
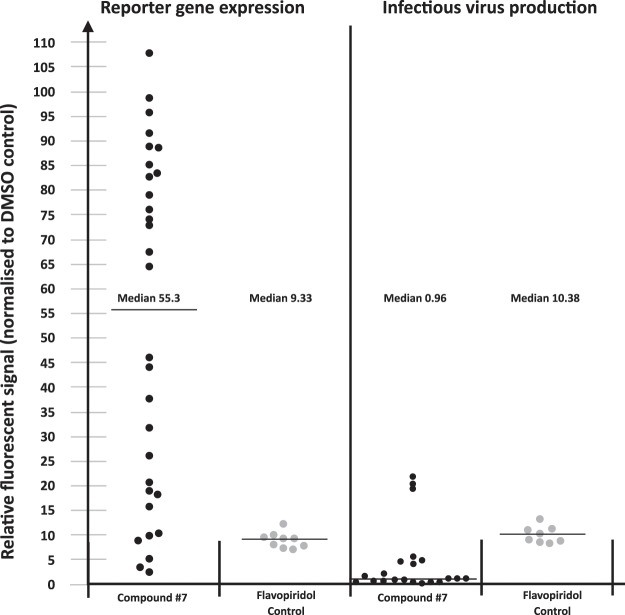
Comparison of effects of compound #7 treatment on reporter gene expression and production of infectious virus in LC5-RIC cultures. LC5-RIC cells contain a reporter gene that is activated by early viral gene expression during HIV infection and encodes a red fluorescent protein (DsRed1). Effects of compound treatment on reporter gene expression and production of infectious virus were determined in LC5-RIC cultures exposed to virus inoculum in the presence or absence of compounds for 48 h. Fluorescent signals of these primary cultures were measured to determine effects on reporter gene expression. Effects on infectious virus production were determined by transferring aliquots of the supernatants of primary cultures to uninfected LC5-RIC cells, and fluorescent signals determined in secondary plates 72 h after transfer. Fluorescent signal intensities of compound-treated cultures were normalized to those of untreated virus-exposed cultures. Compound treatments were performed at 50 nM. Flavopiridol, an inhibitor of HIV transcription, was used as control compound. Data points are from 10 assays performed in triplicate for compound **#7** and 3 assays performed in triplicate for Flavopiridol.

Figure 2 shows data from multiple experiments comparing effects of compound treatment on reporter gene expression and infectious virus production. As expected, treatment with Flavopiridol yielded both very strong inhibition of DsRed1 expression and infectious virus production. In contrast, compound **#7** treatment displayed highly variable effects on DsRed1 expression, whereas it strongly inhibited virus production in all experiments. Indeed, even in experiments in which DsRed1 expression was not or poorly inhibited, compound **#7** strongly blocked infectious virus production. These results indicate that while compound **#7** treatment can have inhibitory effects on early viral gene expression, this inhibitory effect is variable and is thus not the major effect blocking infectious virus production.

**Figure 3:**
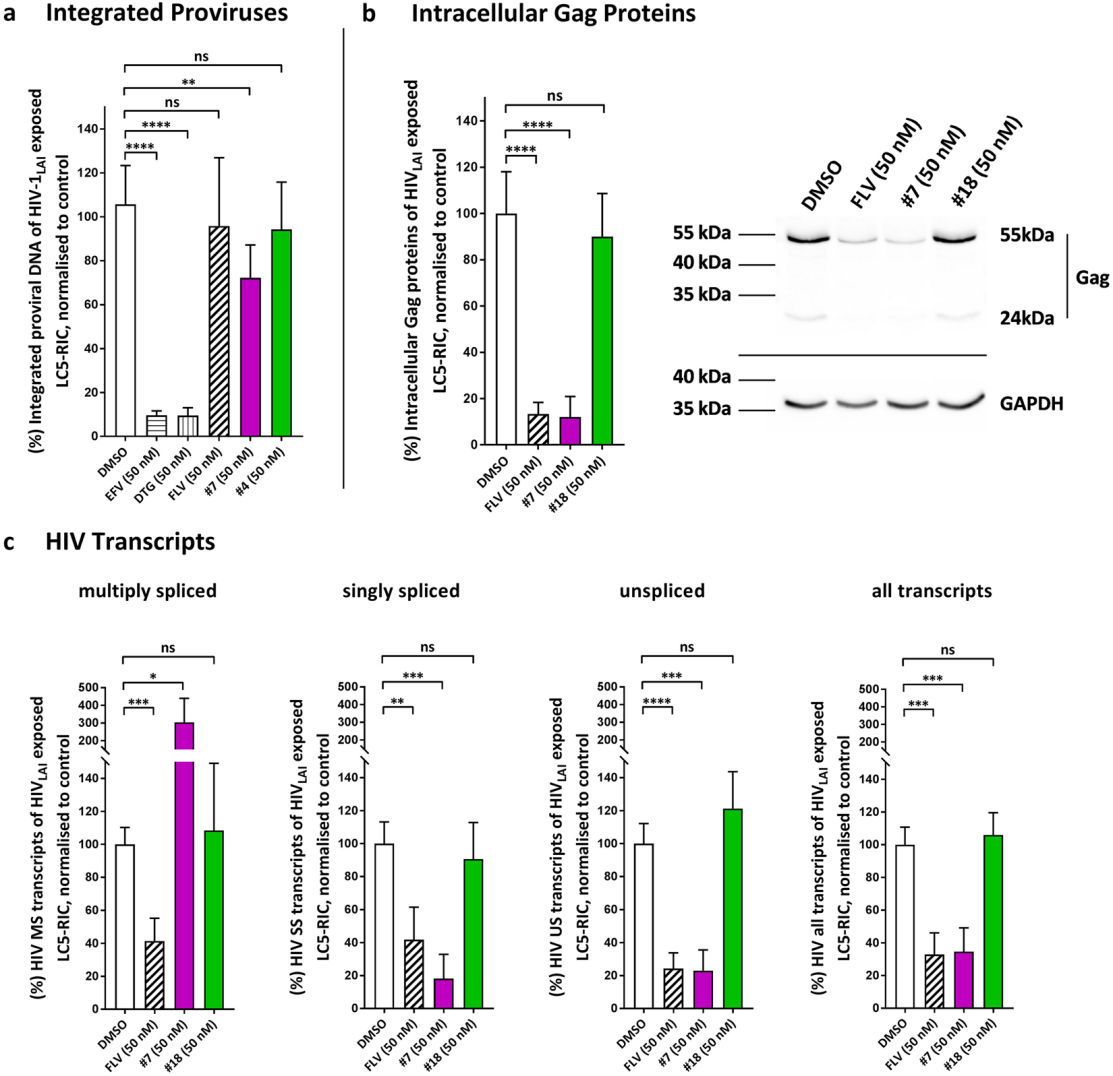
Compound #7 inhibits expression of HIV virion components from integrated proviruses on protein and RNA levels. LC5-RIC cells were exposed to HIV-1_LAI_ and to Aureothin derivatives or to reference compounds or to solvent control (DMSO) for 48 h and subsequently assayed for levels of integrated proviral DNA **(a)**, intracellular Gag proteins **(b)** and different classes of HIV transcripts **(c)**. Aureothin derivatives consisted of the prototypical compound **#7** or the negative control compounds (**#4** or **#18**).The HIV reverse transcriptase inhibitor Efavirenz (EFV), the integrase inhibitor Dolutegravir (DTG) and the HIV transcriptional inhibitor Flavopiridol (FLV) were used as reference compounds. Results from samples exposed to solvent (DMSO) without compound were used for normalization of compound effects. **(a)** Genomic DNA was extracted from cells and used to quantify levels of integrated proviral DNA by Alu-PCR. Shown are the means of three independent experiments with biological triplicates (n = 3; m = 3) and the standard deviation (SD), normalized to DMSO control. **(b)** Samples of cell lysates were evaluated for the presence of Gag by p24-ELISA and by western blot analysis, using antibodies against Gag-p24. GAPDH served as loading control in the western blot. The graph shows the means of three independent experiments with biological duplicates (n = 3; m = 2) and SD, normalized to DMSO control. The western blot is representative for 3 individual experiments. All samples for Gag and GAPDH detection derive from the same experiment; both blots were processed in parallel. Full-length blots are presented in Supplementary Fig. S2. **(c)** RNA was extracted from cells and real-time PCR used to quantify relative levels of the indicated HIV-transcript classes in treated versus untreated cultures. Shown are the means of three independent experiments with biological duplicates (n = 3; m = 2) and SD, normalized to DMSO control. Dunnett’s test was performed to determine whether results from treated samples were significantly different (*P* < 0.05) from the DMSO control. Levels of statistical significance of *P* values are indicated by asterisks, with **P ≤ 0.01, ***P ≤ 0.001, ****P ≤ 0.0001, ns = not significant.

For further analysis of antiviral effects of compound **#7**, we investigated effects of compound **#7** treatment on levels of integrated proviral DNA and cellular viral proteins and mRNAs in LC5-RIC cells, under conditions fully inhibiting HIV production (Fig. 3). Treatments were also performed with compounds **#4** or **#18**, which are at least 400-fold less active against HIV than compound **#7** (Supplementary Table S1) as negative compound controls.

Levels of integrated proviral DNA in HIV-exposed LC5-RIC cultures treated with compound **#7** were evaluated by Alu-PCR. Parallel assays were performed with two reference compounds that prevent HIV integration, the reverse transcriptase inhibitor Efavirenz (EFV), and the integrase inhibitor Dolutegravir (DTG). Treatment with either compound **#7** or with reference drugs was performed at concentrations yielding full inhibition of infectious virus production (>2x IC_90_). Treatment with EFV or DTG strongly reduced levels of integrated proviral DNA (>90%), confirming that our experimental conditions allow detection of compound-mediated reduction of proviral DNA levels (Fig. 3a). Furthermore, treatment with the HIV transcription inhibitor Flavopiridol (FLV) or the control compound **#4** did not reveal reduction of proviral DNA levels, as expected.

Compound **#7** treatment only very moderately affected proviral DNA levels (<20% reduction). This indicates that compound **#7** does not primarily block HIV replication by inhibiting provirus integration.

The viral Gag protein is the major structural protein of HIV virus particles and is produced from integrated proviruses. Therefore, we investigated the effect of compound **#7** treatment on intracellular Gag-levels by p24 ELISA and by western blot analysis of cell lysates with antibodies raised against the Gag p24 protein (Fig. 3b, Supplementary Fig. S2). These results revealed that compound **#7** strongly suppressed production of Gag (Fig. 3b).

HIV expression follows provirus integration in the HIV replication cycle. During expression, multiple mRNAs are generated from a primary transcript by alternative splicing^20^, forming three classes of HIV mRNAs (unspliced, singly spliced and multiply spliced HIV mRNAs; see overview in Supplementary Fig. S3). HIV Gag is produced from unspliced mRNAs, which, together with singly spliced mRNA, predominate in the late phase of HIV gene expression. We determined relative levels of these transcript classes in untreated and compound **#7** treated HIV-exposed LC5-RIC cultures by quantitative real-time PCR (qRT-PCR) analysis with primers specific for each transcript class. As shown in Fig. 3c, levels of unspliced transcripts as well as singly spliced transcripts were strongly diminished in cells treated with compound **#7**, compared to untreated cells. Furthermore, qRT-PCR analysis with primers designed to recognize all transcripts (Fig. 3c) showed that compound **#7** treatment reduced the overall abundance of HIV mRNAs. Interestingly, treatment with compound **#7** did not reduce relative levels of multiply spliced transcripts in these experiments (Fig. 3c). This is in contrast to the effect of Flavopiridol, which showed a much stronger inhibitory effect on levels of multiply spliced transcripts than compound **#7**.

Together these results demonstrate that compound **#7** inhibits the HIV replication cycle after integration of proviruses, diminishing production of unspliced and singly spliced viral transcripts, which are key transcripts for *de novo* virus production.

### Proteome-wide analysis of compound #7 effects in PBMCs

Our next goal was to investigate overall effects of compound **#7** treatment on expression of cellular proteins, both on a general level and in the context of HIV infection.

We carried out semi-quantitative analysis of the proteomes of PBMCs treated with compound **#7**, with or without exposure to HIV (Data provided in Supplementary data file S2). Treatment experiments were performed with PBMC isolates from three donors. Effective inhibition of virus production in compound treated, HIV-exposed samples was confirmed by quantification of infectious virus levels in culture supernatants.

The low proportion of differentially expressed proteins detected for compound **#7** treated samples from each donor (<10%; Supplementary Fig. S4) indicated that HIV inhibition by compound **#7** treatment is not caused by a global effect on cellular protein expression.

Results were individually analysed for significantly changed proteins (Supplementary data file S2) and the significantly changed proteins from all donors were then pooled as biological replicates (separately for up- and down-regulated proteins; Supplementary data file S3).

Genes corresponding to the differentially regulated protein sets were subjected to enrichment analysis to identify overrepresented terms in multiple data bases.

**Figure 4:**
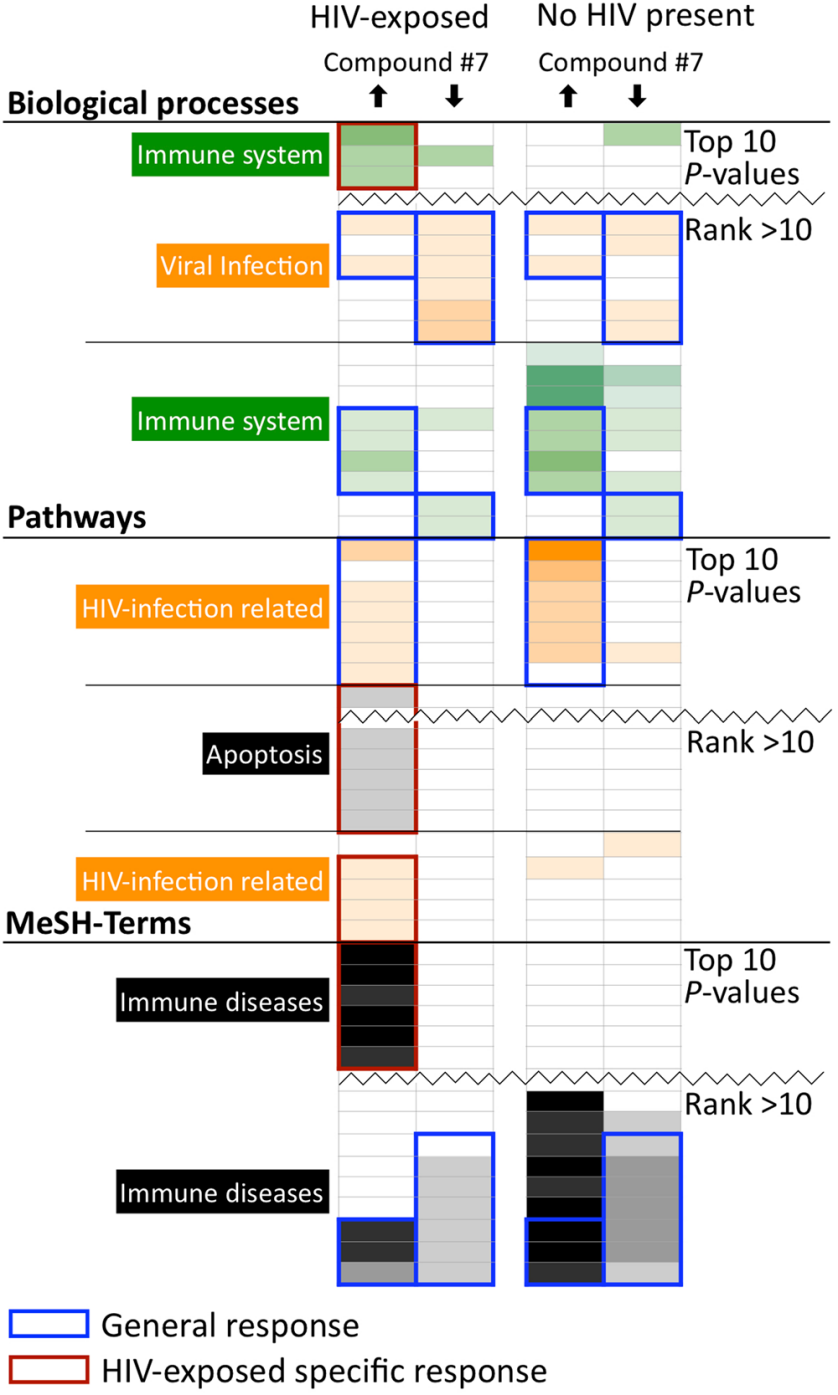
Summarised enrichment analysis profile of proteins differentially expressed in PBMCs as a consequence of compound **#7** treatment. PBMC isolates from three different donors were used as biological replicates and the lists of genes up- or down regulated by treatment with compound **#7** were determined. GO-terms, canonical pathways, and MeSH terms enriched in either HIV-exposed PBMC (↑ up-regulated, ↓ down-regulated) or PBMC exposed to compound **#7** in the absence of HIV (also up- and down-regulated) were determined and shown as heat map. Summary terms are shown color-coded on the left. The heat map is coded by colour saturation (in %): p-value range =% colour saturation: e^−3^ to e^−5^ = 20, e^−6^ to e^−8^ = 40, e^−9^ to e^−11^ = 60, e^−12^ to e^−14^ = 80, ≥e^−15^ = 100. Shared enrichments are boxed in blue, HIV-exposure specific enrichments are boxed in red. More detailed information about terms and proteins are shown in Supplementary Table S3 and Data files S2, S3.

Enrichment analysis of the set of differentially regulated genes revealed overrepresentation of several terms in both HIV-exposed and unexposed gene subsets (Fig. 4 framed in blue; Supplementary Table S3). Enrichments were consistent but small. There were also terms associated especially with HIV-exposure mainly in the subset of down-regulated genes. The highest ranking common pathway terms from the Canonical Pathways database were related to *HIV infection* (Fig. 4; Supplementary Table S3).

In order to address that a majority of uninfected cells might have obscured HIV-infection related proteomics effects we carried out proteome analysis as described for the PBMCs with CD4+ enriched cells (~94% CD4+ cells) from three additional donors. The results were generally similar but showed fewer associated GO-terms and pathways for the infected cells and almost no such enrichment for the uninfected cells (Supplementary Table S3).

In summary, proteomics analysis suggests good biocompatibility of compound **#7** treatment with only limited global effects on protein expression in PBMCs. Profiling these few expression changes by enrichment analysis revealed a set of terms selectively overrepresented in HIV-exposed samples.

### Broad activity of compound #7 against different HIV-genotypes

**Figure 5:**
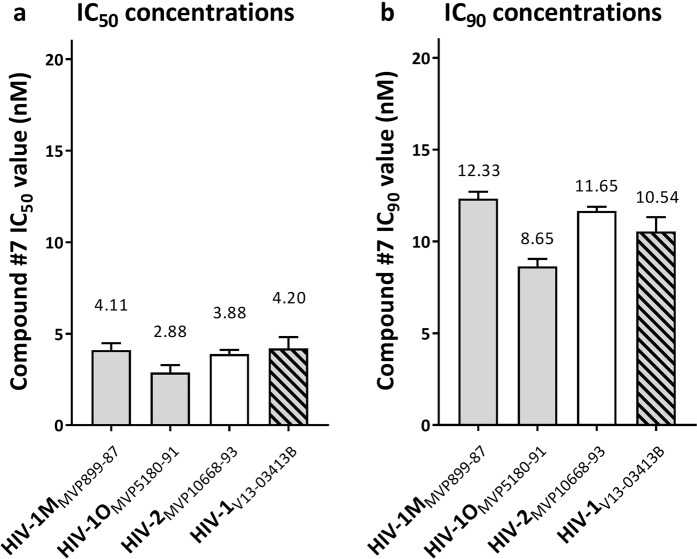
Compound #7 potently inhibits multiple HIV genotypes. PBMCs were exposed to the indicated clinical virus isolates and serial concentrations of compound **#7** and **(a)** half maximal (IC_50_) and **(b)** 90% (IC_90_) HIV-inhibitory concentrations were determined. Clinical isolates represented the following HIV genotypes: HIV-1M_MVP899-87_: HIV-type 1, Major group; HIV-1O_MVP5180-91_: HIV-type 1, Outlier group; HIV-2_MVP10668-93_: HIV-type 2; HIV-1_V13-03413B_: HIV-type 1, Major group/B-clade, isolate with multiple drug-resistance mutations^8^. Depicted are the means of three independent experiments with biological triplicates (n = 3; m = 3) and standard deviations (SD).

To evaluate the inhibitory activity of compound **#7** against different HIV genotypes, we used clinical virus isolates representing the two HIV-types, i.e. HIV-type 1 and HIV-type 2. In addition, HIV-type 1 virus isolates were examined from two groups, i.e. the major group M (HIV-1M_MVP899-87_), and the outlier group O (HIV-1O_MVP5180-91_). Antiviral activities were evaluated in primary human HIV-1 target cells, i.e. PBMCs. As shown in Fig. 5, compound **#7** inhibited replication of all clinical isolates with efficacies similar or even higher than against the prototypical laboratory HIV-1 isolate (HIV-1_LAI_, Table 1).

Finally, we found compound **#7** as a potent inhibitor of an HIV-1 M group/B-clade isolate (HIV-1_V13-03413B_) with multiple drug resistance mutations derived from a patient with virological treatment failure^8^ (Fig. 5).

Together these results demonstrate that compound **#7** displays potent antiviral activity against multiple HIV genotypes and potently inhibits replication of viruses with multiple drug resistance mutations.

### Combination of compound #7 with reverse transcriptase and integrase inhibitors leads to synergistic anti-HIV activity

**Figure 6:**
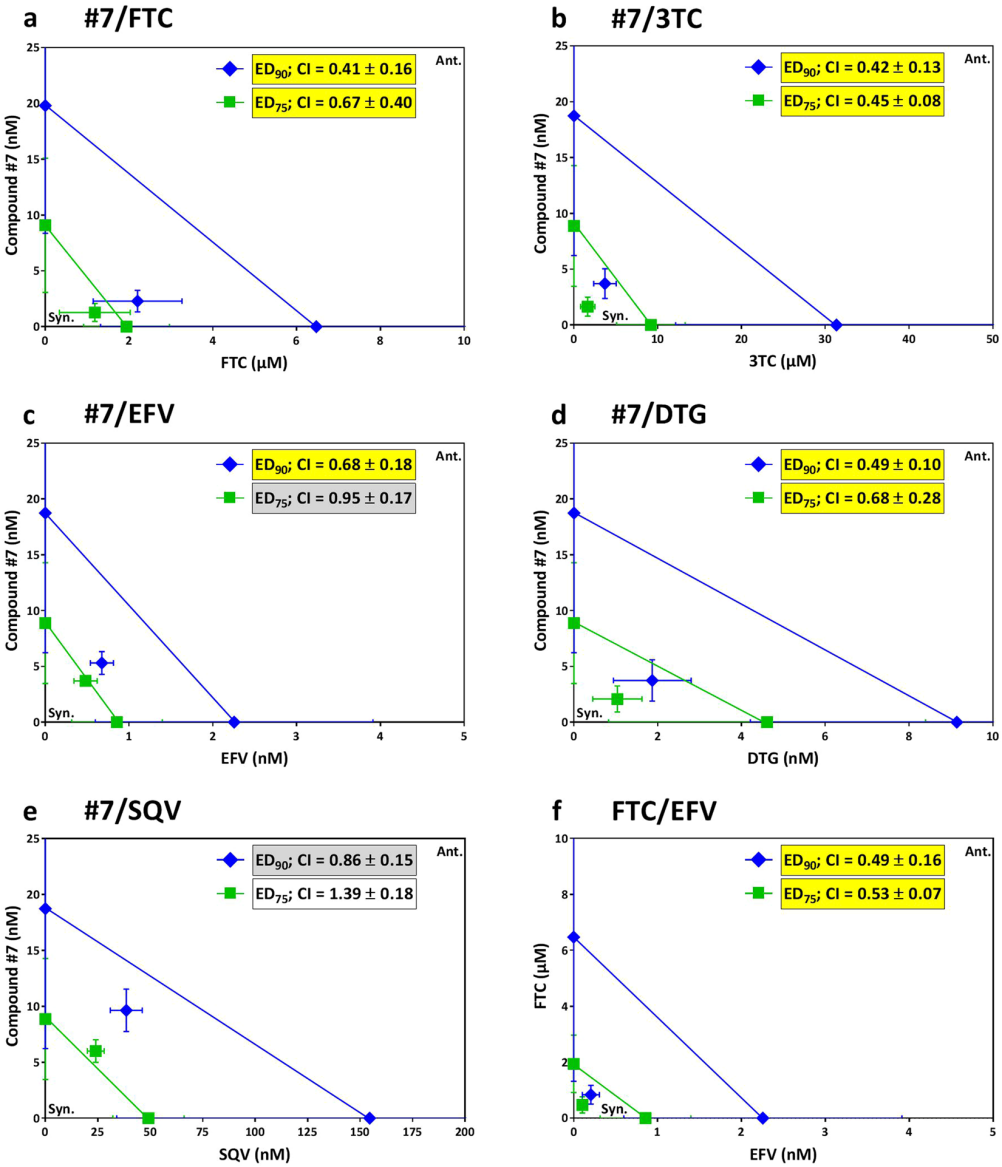
Combinations of compound #7 with reverse transcriptase or integrase inhibitors exhibit synergistic anti-HIV activities. Drug combination effects were determined in LC5-RIC cells exposed to HIV-1_LAI_ and analysed by the Chou-Talalay theorem as described in Materials and Methods. Combinations of compound **#7** with RT inhibitors **(a,b,c**) and integrase inhibitors **(d)** resulted in synergistic inhibition of HIV infection. No synergisms were observed for combination of compound **#7** with a protease inhibitor **(e)**. **(f)** Shows results for combinations of two anti-HIV drugs with reported synergisms for reference. Isobolograms and their combination indices (CI) are shown for 90% (ED_90_) and 75% (ED_50_) HIV inhibition. In the isobolograms, data points that lie to the left of the respective hypotenuse indicate synergism, data points on the hypotenuse additive effects and data points to the right of the hypotenuse antagonism. CI values <0.7 indicate synergistic effects (highlighted yellow), 0.85 to 1.20 additive effects (grey shading) and >1.20 antagonistic effects (no shading)^21,23^. The following clinical drugs were used: RT inhibitors Emtricitabine (FTC; panels a,f), Lamivudine (3TC, panel b), Efavirenz (EFV; panels c,f); integrase inhibitor Dolutegravir (DTG; panel d); protease inhibitor Saquinavir (SQV; panel e). Depicted are the means of at least three independent experiments with biological triplicates (n ≥ 3; m = 3) and standard deviations.

Since current anti-HIV therapies depend on the combined activities of multiple HIV inhibitors, another important aspect in initial assessment of the drug potential of compound **#7** was to demonstrate that it is suitable for combination with selected clinical drugs. Furthermore, the different mode-of-action of compound **#7** from approved anti-HIV drugs (Fig. 3) further supported investigation of occurrence of synergistic effects. Efficacies of HIV inhibition were determined for two-drug mixtures and for single drugs in parallel in HIV exposed cells and combination effects evaluated with the Chou-Talalay theorem^21,22^. Figure 6 shows the combination indices and isobolograms determined for 90% and 75% HIV inhibition (ED_90_, ED_75_), respectively. As assay control we evaluated the combinatorial activities of the reverse transcriptase inhibitors Efavirenz (EFV; NNRTI) and Emtricitabine (FTC; NRTI), which were previously reported to synergistically inhibit HIV replication *in vitro*^23^. Combination data points lying to the left of the hypotenuse and combination indices (CI) <0.7 (Fig. 6) confirmed synergism of these drugs at ED_90_ and ED_75_ under our experimental conditions.

Combinations of compound **#7** with FTC and 3TC, both commonly used reverse transcriptase inhibitors, revealed synergistic effects at ED_90_ and ED_75_. Synergistic effects were also observed for combination of compound **#7** with an integrase inhibitor (DTG). Synergistic effects of compound **#7** and DTG combinations were somewhat lower at ED_75_ than at ED_90_. Synergistic effects were not observed for combinations of compound **#7** and protease inhibitors at either ED_90_ or ED_75_.

These results clearly support the capacity of compound **#7** to inhibit HIV and synergize with selected clinical drugs, including reverse transcriptase inhibitors and integrase inhibitors, in HIV-exposed cells. Thus they encourage future studies addressing the physiological relevance of the synergisms and detailed analysis of potentially complex combinatorial compound effects^24,25^.

## Discussion

In the literature, biological studies of Aureothin addressed mainly its potential to kill microorganisms, parasites and vermin and to inhibit proliferation of selected cancer cell lines^26–30^. We identified the γ-pyrone polyketides Aureothin/Neoaureothin as potent hits during screening of a natural compound library for HIV inhibitors. Only very limited preliminary information supporting antiviral activity of Aureothin is available to date^31^. Our study provides in-depth information on the anti-HIV activity of these γ-pyrone polyketides by identifying a highly active, synthetic variant with improved features, elucidating its mode-of-action in the context of the HIV replication cycle, demonstrating activity against a wide spectrum of HIV genotypes and providing evidence for synergisms with clinical anti-HIV drugs.

We showed that derivatization strategies can be used to improve properties of the Aureothin scaffold impeding drug development (i.e. toxicity, photolability) without eliminating antiviral activity. To this end we generated a collection of fully synthetic Aureothin derivatives, which we gave the acronym MAGIC (Multiple Antiviral Gamma-pyrone Inspired Compounds). These contained various modifications at the aryl moiety designed to replace the potentially toxic nitro group with more favourable substituents like fluorine. Selective introduction of fluorine residues has been used to improve biological properties of numerous drug candidates^32^ and many clinical anti-HIV drugs contain one or more fluorine atoms (Emtricitabine, Raltegravir, Dolutegravir, Efavirenz).

Parallel profiling of cell tolerance and anti-HIV activity in PBMCs showed improved cell tolerance for all derivatives compared to Aureothin. Several fluorine-containing derivatives were identified with similarly potent anti-HIV activity as Aureothin (**#6**, **#7**, **#14**). Particularly compound **#7**, which contains a trifluoromethyl- replacing the nitro-group, was most effective in achieving>90% inhibition of HIV infection in PBMCs (IC_90_ <45 nM). Substitutions by chlorine (compound **#3**, **#13**) resulted in weaker compounds (IC_90_ ~2000 to 3000 nM).

Several derivatives showed better photostability than Aureothin and several molecules with fluorine or chlorine substituents retained over 95% of anti-HIV activity after illumination. Improved photostability of the Aureothin scaffold apparently can be achieved by introduction of halogens and is not limited to fluorine residues.

Overall these results confirm that the Aureothin scaffold can be improved without perturbing antiviral activity and indicate that different determinants of the Aureothin scaffold direct antiviral activity and properties important for drug development like cell tolerability and light sensitivity.

**Figure 7:**
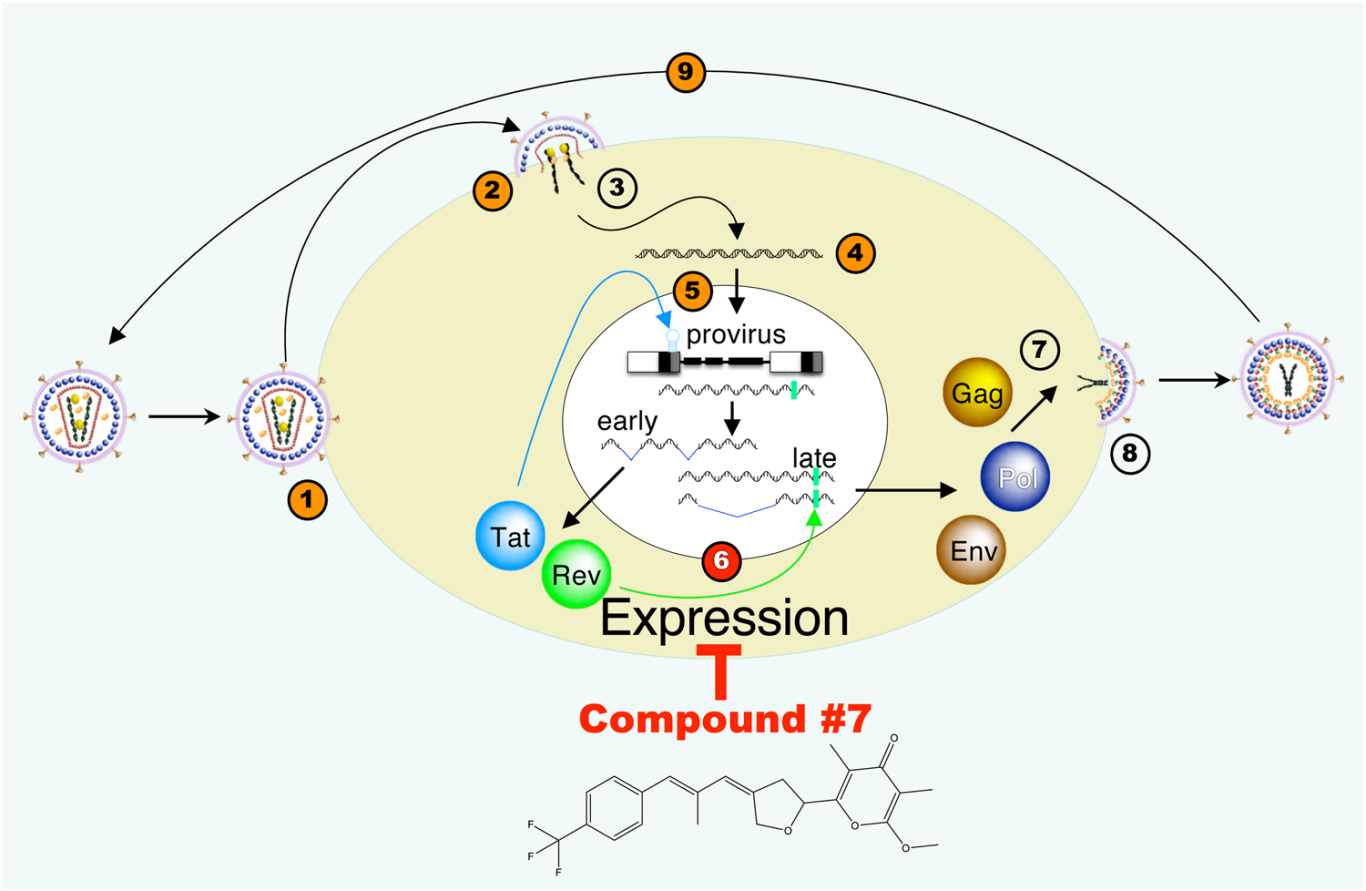
Mode-of-action of compound **#7** in the HIV replication cycle: blocking of *de novo* virus production by inhibiting HIV gene expression. The HIV replication cycle is shown with numbers indicating the consecutive steps of the replication cycle. 1, Attachment of virus to receptor; 2, Fusion of viral and cellular membranes; 3, Uncoating and delivery of viral RNA to cytoplasm; 4, Generation of the DNA copy of viral genome by reverse transcription; 5, Nuclear import of viral DNA and integration into host cell genome; 6, Virus expression; 7, Assembly of Gag, Pol, Env proteins and unspliced viral RNAs to virus particles; 8, Budding of immature virus particles; 9, Maturation of virus particles. Steps of the replication cycle attacked by clinically approved drugs are indicated by circles with orange fill colour (Steps 1, 2, 4, 5, 9). Compound **#7** inhibits virus expression (6, red fill colour), which is not blocked by current clinical drugs. The mRNAs suppressed by compound **#7** encode for Gag, Pol, Env proteins and included viral RNAs packaged into new virions.

We chose compound **#7** based on high antiviral potency, improved cell tolerance and photostability as a lead candidate for further analysis of this compound class. Compound **#7** treatment only very moderately affected proviral DNA levels, pointing to post-integration steps of the HIV replication cycle as targets for compound **#7**. *De novo* virus production depends on accumulation of unspliced Gag-encoding transcripts as well as partially spliced Env-encoding transcripts during the late phase of HIV expression^20^. We show that compound **#7** very strongly reduced levels of the viral Gag polyprotein. Importantly, compound **#7** treatment inhibited accumulation of both unspliced and partially spliced mRNA, preventing the production of all major components of virus particles. The mode-of-action of compound **#7** is different from all current clinical anti-HIV drugs (Fig. 7)^33^.

Compound **#7** treatment affected levels of unspliced and partially spliced HIV transcripts much more strongly than those of multiply spliced transcripts. Thus, effects of compound **#7** treatment on HIV transcripts were different from those of the HIV transcriptional inhibitor Flavopiridol^18,19^, which reduced levels of all three HIV transcript classes to similar extents. This suggests that compound **#7** affects post-transcriptional events of HIV expression involved in the production and accumulation of unspliced and multiply spliced transcripts. These post-transcriptional processes include alternative splicing of the HIV pre-mRNA, stabilization and nuclear export of intron-containing HIV mRNAs and their translation^34–39^ and involve numerous interactions of viral components with cellular partners. We expect future studies dissecting the mechanisms underlying the novel mode-of-action of compound **#7** to identify key cellular pathways of HIV expression that can be exploited for anti-HIV drug development.

Compound **#7** affects only a small part of the proteome of PBMCs (Supplementary Fig. S4), confirming that the inhibition of viral protein expression is not a consequence of unspecific global inhibition of cellular protein expression. This was also observed for CD4+ enriched cells (Supplementary Table S3). Enrichment analysis of GO-, MeSH-terms and pathways identified various terms connected with the immune system and with viral (including HIV) infection (Fig. 4). Several terms were enriched only in HIV-exposed compound **#7** treated cultures. However, the overall results indicate that whole proteomics analysis, by itself, is not sufficient for identification of specific signalling pathways and single genes targeted by compound **#7**. Thus the effect of compound **#7** on cellular processes specific for HIV-exposed human primary blood cells awaits further elucidation, possibly by combining results from multiple systems approaches.

In this study we show that compound **#7** effectively inhibits a spectrum of clinical isolates that includes all HIV genotypes (HIV-type 1 and HIV-type 2) (Fig. 5), including a virus isolate with multiple drug resistance mutations. The broad and potent inhibitory activity of compound **#7** against multiple HIV genotypes is in line with a cell-focused mode-of-action. In contrast, some compounds designed to interfere with viral components show selective inhibition of individual HIV genotypes. For example, the HIV fusion inhibitor Enfuvirtide and the NNRTI Efavirenz, standardly used for treatment of HIV-1, are ineffective against HIV-2 because the virus is intrinsically resistant to these compounds^40,41^. This suggests a therapeutic potential of compound **#7** for treatment of HIV-2 infections, which often remain undetected and may not be effectively treated with regimens designed for treatment of HIV-1.

The standard anti-HIV drug therapy involves the combination of drugs from different classes that target at least two different steps of the HIV replication cycle^1^. Since compound **#7** acts on a step different from all known drugs it is an excellent candidate for combination anti-HIV therapy. In support, we demonstrate its capacity to synergize with several clinical anti-HIV drugs.

We conclude that the compounds described here represent a promising new class of powerful HIV inhibitors that are chemically and functionally different from all current clinical anti-HIV drugs. The prototypical compound **#7** inhibits HIV gene expression, with strong effects on production of components of virus particles, thus interfering with a stage of the virus replication cycle not attacked by clinical drugs so far. Proteomics analysis of treatment effects on PBMCs indicated no general disturbance of cellular protein expression, and revealed moderate HIV-selective responses. In addition we show that these compounds can synergize with selected clinical drugs in cell culture experiments and that they are active against multiple HIV genotypes. Future studies will be directed towards the use of these compounds to identify virus-host interactions that represent novel points of attack for HIV inhibition as well as further exploration of the potential of these compounds for development into drugs.

## Methods

### Viruses and virus production

HIV-1 stocks of the laboratory isolate HIV-1_LAI_ were produced by transfection of HEK293T cells with the proviral plasmid pLAI.2 (NIH AIDS Research and Reference Reagent Program, Division of AIDS, NIAID, NIH, from Dr. Keith Peden^42^) as described^8^.

Isolation of primary clinical HIV-isolates HIV-1M_MVP899-87_, HIV-1O_MVP5180-91_, HIV-1_V13-03413B_ and HIV-2_MVP10668-93_ is described in^43^. The HIV-1 M group/B-clade isolate (HIV-1_V13-03413B_) was isolated from a patient with virological treatment failure^8^. Clinical isolates were produced from HIV infected H9 cells as described^8^.

Infectious titres of virus samples were determined by measuring the Tissue Culture Infectious Dose 50 (TCID_50_), using LC5-RIC HIV reporter cells. Briefly, 10,000 LC5-RIC reporter cells per well were plated into black 96-well plates, incubated for 24 h and inoculated with serial dilutions of the virus sample (initial dilution 1:6; 11 dilution steps of 1:5, six replicates of each dilution tested). Plates were incubated for 48 h; wells were visually inspected for cells expressing the fluorescent reporter DsRed1 by fluorescence microscopy. Wells with at least one DsRed1-positive cell were counted positive. TCID_50_ was calculated as described by Reed^44^.

### Cells and cell culture

HEK293T (ATCC®-Number CRL-11268), the HIV reporter cell line LC5-RIC, the human T-lymphoma cell line H9 (ATCC®-Number HTB-176) and peripheral blood mononuclear cells (PBMC) were cultured as described in^6,8^. PBMCs were isolated from buffy-coats (received from the Blood Donation Services of the Bavarian Red Cross GmbH) by Ficoll density gradient centrifugation as described in^8,45^. Prior to infection, PBMCs were stimulated with 20 U/ml hIL-2 and 1 µg/ml phytohemagglutinin (PHA) for 72 h. CD4+ cells were enriched from whole blood using the RosetteSep^TM^ Human CD4+ T Cell Enrichment Cocktail from Stemcell Technologies according to the manufacturer's instructions and cells handled like PBMCs. CD4+ cell enrichment was verified by staining with APC mouse anti-human CD4 antibody (BD Pharmingen) and FACS analysis, yielding ~94% CD4+−positive cells.

All cells were cultured under standard conditions, 37 °C and 5% CO_2_.

### Aureothin and derivative

Aureothin (BVT-0303-M001) was purchased from Biomol. All Aureothin derivatives were produced by total synthesis by Bicoll Biotechnology Co. Ltd., mainly following the synthesis scheme of^14,15^.

^1^H-NMR spectra of all derivatives are shown in Supplementary Data File S1.

### Reference HIV-1 inhibitors

Enfuvirtide (T-20; SML0934) was purchased from Sigma-Aldrich, Emtricitabine (FTC; ab145045) from abcam, Abacavir (ABC; Cay14746-10) from Biomol, Lamivudine (3TC; T0682) and Dolutegravir (DTG; T6198) from TargetMol, Efavirenz (EFV; 14412) and Saquinavir (SQV; 9001893) from Cayman.

### Cell viability and toxicity assays

The following assays were used to profile cell safety of compound treatment in HIV-exposed cultures (LC5-RIC or PBMC): (1) CellTox^**TM**^ Green Cytotoxicity Assay, Promega, measures binding of a fluorescent dye to DNA of cells with impaired membrane integrity; (2) CellTiter-Glo^®^ 2.0 Assay, Promega, which measures the amount of ATP in viable cells after lysis using an assay with luminescence read-out. Cells were plated into black 96-well plates followed by overnight incubation at 37 °C, 5% CO_2_. Compounds dissolved in DMSO were screened at multiple concentrations from 0.6 to 10,000 nM at a final DMSO concentration of 0.04% followed by additional 48 h incubation. Both assays were performed according to manufacturer’s recommendations and consecutively in the above mentioned order. Cells were washed once with PBS between the assays.

### Cell-based assays for characterization of antiviral activities

#### Inhibition of infection assays

Anti-HIV activities of compounds were profiled in LC5-RIC cells with the EASY-HIT assay as described in^6^. Briefly, LC5-RIC cells were first exposed to virus and compounds (first step of the assay). Compound effects on infectious virus production were determined by transferring aliquots of supernatants of compound-treated cultures to secondary plates and measurement of fluorescent signal intensities in secondary plates after 72 h (second step of the assay). Fluorescent signal intensities determined for compound-treated cultures were normalized to those determined for untreated virus-exposed cultures. Cells were exposed to the designated virus strain at an MOI of 0.5.

To evaluate anti-HIV efficacies of test compounds in primary human target cells, stimulated PBMCs were seeded in 96-well plates at a density of 500,000 cells per well, different #7concentrations of the compound (serial dilutions) and virus inoculum (MOI of 0.5) added to the PBMCs and cultures incubated for 48 h. Infectious virus production was quantified by transferring 35 µl of supernatant from the PBMC cultures to LC5-RIC cells, seeded in black 96-well plates one day earlier. LC5-RIC cultures were then incubated for 72 h and analysed for DsRed1 reporter expression and anti-HIV activity calculated as described for the EASY-HIT assay.

#### Evaluation of photostability of antiviral activities of Aureothin-derivatives

LC5-RIC cells were plated as described for the EASY-HIT assay. 24 h previous to treatment and inoculation of the cells, compounds (in DMSO) were diluted in cell culture medium to their IC_90_ concentrations and applied to 96-well plates (triplicate wells for each compound). Plates were incubated either in the dark or under constant illumination with a commercially available desk lamp (2X Philips Master PL-S-9W/840/2 P, 30 cm between light source and compounds) for 24 h at room temperature. For analysis of anti-HIV activity, compound samples were transferred to LC5-RIC cells and their anti-HIV activity determined by measuring compound effects on infectious virus production with the EASY-HIT assay. Relative photostability was calculated by normalizing the anti-HIV activity of the illuminated compounds to the antiviral activity of compounds kept in the dark.

### Assays for quantification of cell-associated HIV infection parameters

*Quantitative analysis of integrated copies of HIV proviral DNA by Alu-PCR* was performed essentially as described in^46^. Briefly, LC5-RIC cells seeded at a density of 100,000 cells per well in 12-well plates and incubated for 24 h were exposed to test compounds and HIV-1_LAI_ virus inoculum at an MOI of 0.5 and incubated for further 48 h at 37 °C, 5% CO_2_. Subsequently, cells were harvested and genomic DNA (gDNA) was extracted using the QIAamp DNA Mini Kit (QIAGEN) according to the manufacturer’s protocol. The relative amount of integrated proviral HIV-1 DNA was determined by two-step Alu-PCR^46^. The relative amounts of integrated proviral HIV-1 DNA in treated versus untreated samples were calculated by the 2^(−ΔΔCt)^ method^47^, with ΔC_T_ values representing C_T_ (HIV) - C_T_ (β-globin) for each sample and ΔΔC_T_ values representing values of treated samples normalized to untreated samples.

#### Quantitative analysis of relative levels of HIV-1 transcripts by qRT-PCR

LC5-RIC cells were plated, treated with compounds and inoculated with virus as described for Alu-PCR above. Cells were then harvested and RNA was extracted using the RNeasy Mini Kit (QIAGEN), according to the manufacturer’s protocol. For cDNA synthesis, 1 µg of total RNA was reverse transcribed by SuperScript II reverse transcriptase (Invitrogen) according to manufacturer’s recommendations. qRT-PCR was performed with the Roche Light Cycler^®^ 480II 96 (Version 1.5.0.39), using LightCycler^®^ 480 SYBR Green I Mastermix (Roche). Quantitative PCR for HIV-1 transcripts was performed with primers specific for unspliced, singly spliced or multiply spliced HIV-1 transcripts as well as for ‘all HIV transcripts’; RNA Polymerase II transcripts were analysed as internal reference gene. Primer sequences are given in Supplementary Methods and binding sites shown in Supplementary Fig. S3. Data was analysed with the second derivative maximum method. The relative amount of HIV-1 transcripts in treated versus untreated samples was calculated by the 2^(−ΔΔCt)^ method^47^, using RNA polymerase II as reference gene.

#### Quantification of Gag-Production

LC5-RIC cells were plated, treated with compounds and inoculated with virus as described for Alu-PCR above. Cells were harvested, washed with PBS and incubated in 200 µl of PBS + 5% Triton X-100 for 5 min at room temperature. Cell lysates were centrifuged for 5 min at 400 g, supernatants collected, diluted 1:10,000 and Gag-p24 was detected by p24 Antigen Capture Assay (ABLinc) according to the manufacturer’s protocol.

Western blot analysis for Gag-p24 and GAPDH was performed as described in^48^, using the primary antibodies anti-hGAPDH (ThermoFisher Scientific) and anti-HIV-p24 (Calbiochem) as well as the secondary anti-mouse-peroxidase antibody (Jackson ImmunoResearch), all diluted 1:10,000. Bands were visualized by enhanced chemiluminescence and detected by Fusion FX Chemiluminescence detector. Resulting blot images were processed with Fusion FX Software (adjusting brightness and contrast) and Microsoft PowerPoint (labelling and cropping).

### Proteomics analyses

Stimulated PBMCs or CD4+ cells (5,000,000 cells) were seeded into 12-well plates before compound treatment and inoculation with HIV-1_LAI_ at an MOI of 0.5, followed by 48 h incubation at 37 °C, 5% CO_2_. HIV inhibition was confirmed by quantifying infectious virus production in LC5-RIC cells as described above. Protein lysates were prepared from cells harvested, washed with PBS and lysed by incubating the cells for 10 min at 4 °C in lysis buffer (100 mM Tris-HCl pH 8.3, 150 mM NaCl, 1% glycerol, 1% NP-40, 0.5% deoxycholate, 0.1% SDS, 0.1% Triton-X100) and centrifuged for 20 min at 4 °C, 16,000 g to clear protein lysates. Protein concentrations were determined by using the Pierce^TM^ BCA Protein Assay Kit (ThermoFisher Scientific) according to manufacturer’s protocol. The proteomes of PBMCs from three different donors were analysed by LC-MSMS and data enrichment analysis was carried out using GeneRanker (Intrexon). For detailed information see Supplementary Methods.

### Analysis of combinatorial anti-HIV activities of compounds

Anti-HIV activity of either single or combination treatments was determined based on the EASY-HIT assay as described above with the following aberrations: Cells were treated with 2-fold serial dilutions (12 different concentrations tested in triplicate wells) of either single compounds or compound combinations at starting concentrations >IC_90_ in both single and combination treatment. To evaluate the interaction between combined compounds, data was analysed using the CompuSyn software that is based on the combination index theorem of^21,22^.

### Statistical analysis

IC_50_/CC_50_ values were calculated with GraphPad Prism v7, using the equation for sigmoidal dose-response with variable slope and constraints set to 0 for bottom and 100 for top. Statistical significance was computed by performing a Dunnett’s test.

## Supplementary information

Supplementary Information.

Dataset 1.

Dataset 2.

Dataset 3.

## Data Availability

All datasets generated during and/or analysed during the current study are available from the corresponding author on reasonable request or are included in this published article (and its Supplementary Information files).
