# Supplementary material for: Potent inhibition of HIV replication in primary human cells by novel synthetic polyketides inspired by Aureothin
Source: Sci Rep. 2020 Jan 28;10:1326. doi: 10.1038/s41598-020-57843-9 (PMC6987146; doi:10.1038/s41598-020-57843-9)
Supplement: Supplementary file 1 — Supplementary Information. [file 41598_2020_57843_MOESM1_ESM.docx]

Potent inhibition of HIV replication in primary human cells by novel synthetic polyketides inspired by Aureothin

Alexander Herrmann^1^, Manfred Roesner^2^, Thomas Werner^3^, Stefanie M. Hauck^4^, Alisha Koch^1^, Amelie Bauer^1^, Martha Schneider^1^, Ruth Brack-Werner^1,*^

^1^Institute of Virology, Helmholtz Zentrum München - Deutsches Forschungszentrum für Gesundheit und Umwelt GmbH, Neuherberg, Germany.

^2^mroe-consulting, Eppstein, Germany.

^3^Department of Computational Medicine and Bioinformatics & Department of Internal Medicine, University of Michigan, Ann Arbor, Michigan, USA.

^4^Research Unit Protein Science, Helmholtz Zentrum München – Deutsches Forschungszentrum für Gesundheit und Umwelt GmbH, Munich, Germany.

^*^Address correspondence to Ruth Brack-Werner, brack@helmholtz-muenchen.de

Supplementary Information

Supplementary Methods

**Aureothin derivative #7**

*Compound* ***#7****:* 4-Trifluoromethylbenzaldehyde was converted with tetrabromomethane and triphenylphosphin/Zn to 2,2-dibromovinyl-4-trifluoromethylbenzene. This was reacted with the boronic ester of the key intermediate γ-pyrone-furanon (compound **#2**). E- and Z-isomers were separated by MPLC chromatography (petroleum ether/ethyl acetate). The Z-isomer (major component) underwent bromo-methyl conversion with Zn(CH_3_)_2_ and Pd-phosphin catalyst to yield (±)-2-Methoxy-3,5-dimethyl-6-((*Z*)-4-((*E*)-2-methyl-3-(4-(trifluoromethyl)phenyl)allylidene)tetrahydrofuran-2-yl)-4*H*-pyran-4-one as a yellow powder.
Molecular formula: C_23_H_23_F_3_O_4_; MW: 420.4; ESI-MS: M^+^+1 = 421.2
^1^H-NMR (400 MHz, CDCl_3_): δ_H_ 1.87 (s, 3H), 2.02 (s, 3H), 2.05 (s, 3H), 2.94 (br, dd, J= 4, 16 Hz, 1H), 3.06 (br, dd, J= 4, 16 Hz, 1H), 3.96 (s, 3H), 4.75 (br, d, J = 4, 16 Hz, 1 H), 4.87 (br, d, J = 16 Hz, 1H), 5.15 (t, J = 4 Hz, 1H), 6.19 (br, s, 1H), 6.35 (br, s, 1H), 7.35 (d, J = 8 Hz, 2H), 7.60 (d, J = 8 Hz, 2H).

**Quantitative analysis of relative levels of HIV-1 transcripts by qRT-PCR**

The following primers were used to perform the qRT-PCR analysis (primer sequences 5’ 🡪 3’): multiply spliced, forward: ATG GAG CCA GTA GAT CCT AG, reverse: AGT CTC TCA AGC GGT GGT ^1^; singly spliced, forward: GGc GGC GAC TGG AAG AGG C, reverse: CTA TGA TTA CTA TGG ACC ACA ^1^; unspliced, forward: CTG AAG CGC GCA CGG CAA, reverse: GAC GCT CTC GCA CCC ATC TC ^1^; ‘all HIV transcripts’, forward: GTG TGT GGT AGA TCC ACA GAT CAA GG, reverse: CCA GTC ACA CCT CAG GTA CCT TTA AGA CC ^2^; RNA polymerase II, forward: GCA CCA CGT CCA ATG ACA T, reverse: GTG CGG CTG CTT CCA TAA ^3^. See primer binding sites in different HIV transcripts in Fig. S1.

**Proteomics analyses**

After measuring the protein concentration of the cell lysates equal total protein amounts (10 µg per sample) were proteolysed with trypsin as described ^4^ by a modified filter-aided sample preparation (FASP) procedure ^5^.

LC-MSMS analysis was performed on a QExactive HF mass spectrometer (ThermoFisher Scientific) online coupled to an Ultimate 3000 RSLC nano-HPLC (Dionex). Samples were automatically injected and loaded onto the C18 trap column and after 5 min eluted and separated on the C18 analytical column (Acquity UPLC M-Class HSS T3 Column, 1.8 µm, 75 µm x 250 mm; Waters) by a 90 min non-linear acetonitrile gradient at a flow rate of 250 nl/min ^6^. MS spectra were recorded at a resolution of 60,000 and after each MS1 cycle, the 10 most abundant peptide ions were selected for fragmentation. Acquired raw data was loaded into Progenesis QI software for proteomics for MS1 intensity based label-free semi-quantification (v3.0, Nonlinear Dynamics, Waters), separately for the different donors and analyzed as described ^6^. MSMS spectra were exported and searched against the SwissProt Human database spiked with sequences for HIV LAI isolate (20,221 sequences) using the Mascot search engine (version 2.6.2). Search settings were: enzyme trypsin, 10 ppm peptide mass tolerance and 0.02 Da fragment mass tolerance, one missed cleavage allowed, carbamidomethylation was set as fixed modification, methionine oxidation and asparagine and glutamine deamidation were allowed as variable modifications. A Mascot-integrated decoy database search was performed with an average false discovery rate of <1% using the mascot percolator algorithm ^7^. Peptide assignments were re-imported into the Progenesis QI software. The abundances of all unique peptides allocated to each protein were summed up. The resulting normalized abundances of the individual proteins were used for calculation of fold-changes of proteins and significance values p by a Student’s T-test followed by FDR correction resulting in given q-values.

*Data analysis.* The results from three donors were regarded as biological replicates. Significantly changed proteins (≥2.0-fold change in expression up or down) were collected separately (for each sample and for up- resp. downregulated proteins). Proteomics results were graphically represented by Volcano Plots generated using Microsoft Excel 2016 with an in-house developed Add-In. Parameters: p-value significance level 0.05; Fold change 2.

The lists for up- resp. downregulated proteins of the three donors were then combined and their corresponding ENTREZ geneIDs were used for further analysis. All together this resulted in four distinct protein/gene lists: Up- and down-regulated proteins/genes derived from HIV-exposed PBMCs and up- and down-regulated proteins/genes from PBMCs not exposed to virus.

Enrichment analyses were carried out using GeneRanker (Intrexon) and significant results were filtered for HIV/retrovirus infection/Immune system related terms (arbitrary term selection). Analyses were carried out separately for GO-terms in ‘biological processes’, canonical pathways, MeSH-terms and unigene tissues.

**Biochemical assays for evaluation of activities on single HIV enzymes.**

*HIV Reverse Transcriptase Activity Assay.* To determine the inhibitory effect of test compounds against HIV reverse transcriptase activity, the colorimetric Reverse Transcriptase Assay Kit (Roche) was performed according to manufacturer’s protocol using 4 ng recombinant HIV-1 reverse transcriptase. Efavirenz was used as reference compound.

*HIV Integrase Activity Assay.* To evaluate the inhibitory effect of test compounds to counteract HIV integrase activity, the HIV-1 Integrase Assay Kit (XpressBio) was conducted according to manufacturer’s recommendations. Dolutegravir was used as reference inhibitor.

*HIV Protease Activity Assay.* 100 ng recombinant HIV-1 Protease protein (Abcam) was incubated with compounds diluted in HIV Protease Activity Assay Buffer (0.1 M sodium acetate, 1.0 M sodium chloride, 1.0 mM ethylenediaminetetraacetic acid (EDTA), 1.0 mM dithiothreitol (DTT), 10% dimethyl sulfoxide (DMSO), 1 mg/ml bovine serum albumin (BSA), pH 7.4) for 1 h at room temperature in the dark. After the designated incubation time the mixtures were added to a 2.5 µM solution of HIV Protease Substrate 1 (Sigma-Aldrich) in HIV Protease Activity Assay Buffer prepared in black 96-well plates. The substrate harbours the fluorophore EDANS (5-(2-aminoethylamino)-1-naphthalene sulfonate) and the acceptor chromophore DABCYL (4’-dimethylaminoazobenzene-4-carboxylate) on opposite sides of the cleavage site (Tyr-Pro). Cleavage of the substrate by the HIV-1 protease results in separation of DABCYL and EDANS, eliminating the quenching effects on EDANS fluorescence at 490 nm. After an additional incubation time of 20 min at 37 °C the increase in fluorescence was measured using a fluorescence microplate reader at an excitation filter wavelength of 340 nm and an emission filter wavelength of 490 nm. Saquinavir was used as reference compound.

**Supplementary Figures and Tables**


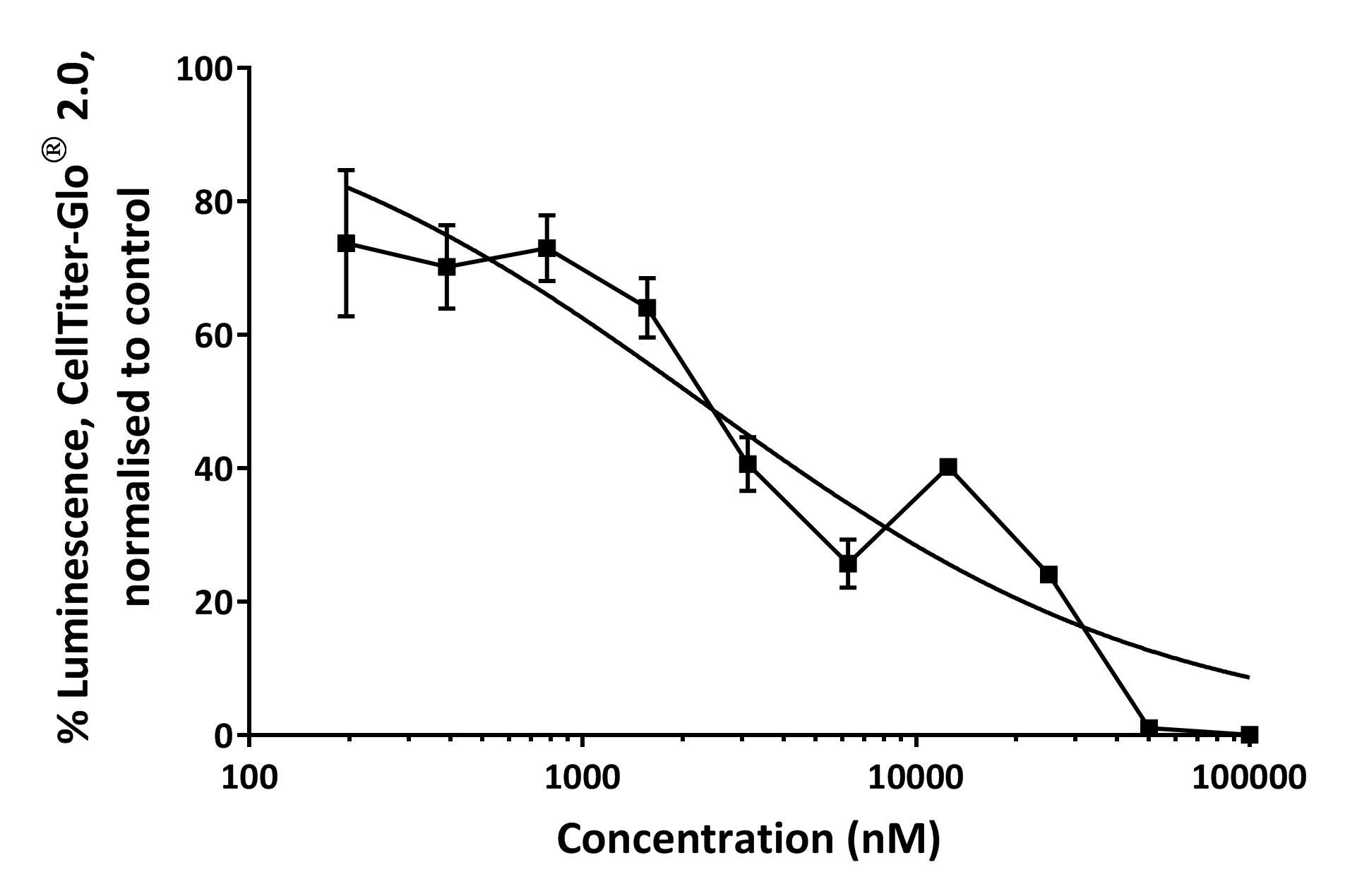


**Fig. S1. Evaluation of cell viability of Aureothin in PBMCs by CellTiter-Glo 2.0 Assay.** Cell viability of PBMCs after treatment with different concentrations of Aureothin was evaluated performing a CellTiter-Glo 2.0 Assay. Depicted are means of triplicates of each concentration. Non-linear regression curve and CC_50_ value (2,271 nM) was calculated using the equation for sigmoidal dose-response with variable slope and constraints set to 0 for bottom and 100 for top.

**
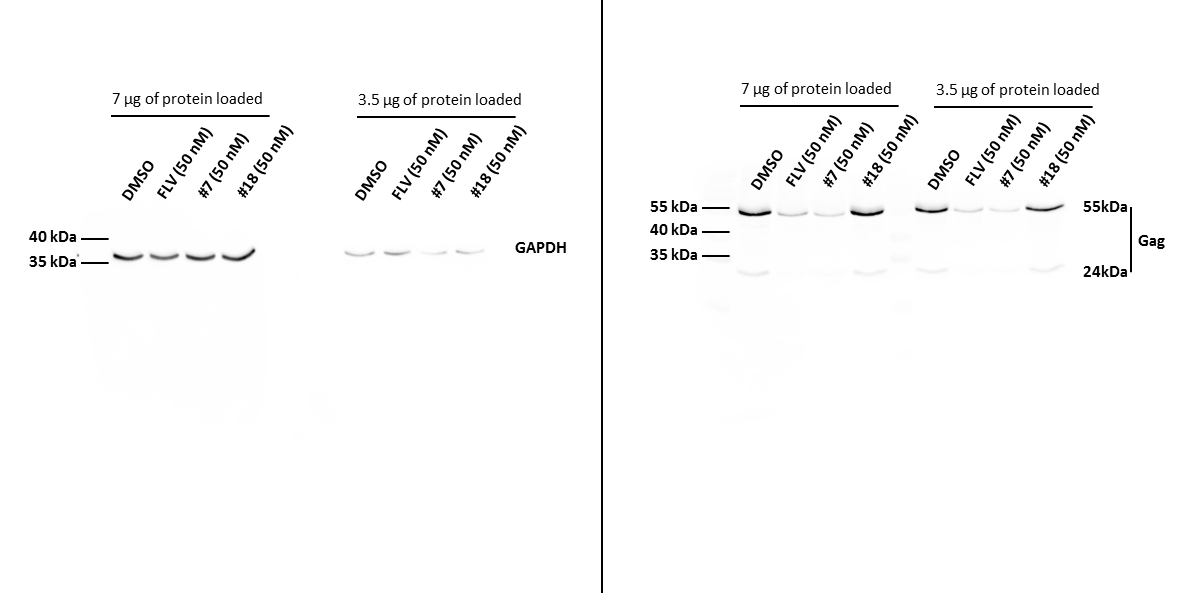
**

**Fig. S2. Full-length western blots used to detect Gag protein and GAPDH.** Cell lysates were evaluated for levels of Gag proteins and GAPDH by Western blot analysis. The full-length blots of GAPDH (left) and Gag proteins (right) show eight lanes in total; 4 lanes each for the conditions (from left to right) DMSO, Flavopiridol (FLV, 50 nM), **#7** (50 nM) and **#18** (50 nM) blotted with two different amounts of protein (7 µg and 3.5 µg). The blots were cropped for the main manuscript (Fig. 3b) by excluding the lanes with 3.5 µg of loaded protein. The Western blots are representative for 3 individual experiments. All samples for both Gag proteins and GAPDH detection derive from the same experiment; both blots were processed in parallel.

**
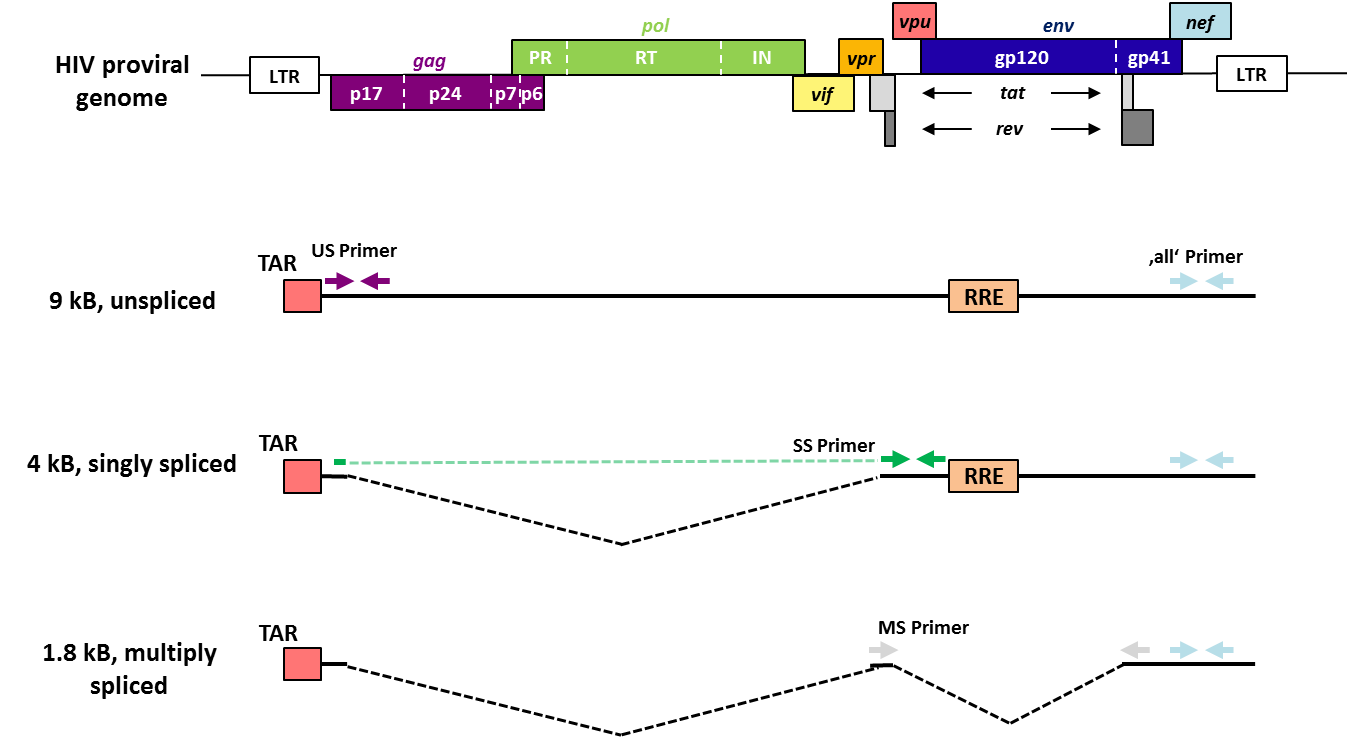
**

**Fig. S3. Overview of HIV proviral genome and HIV transcript classes and the binding sites of primers used for RTqPCR.** Due to extensive alternative splicing during HIV expression there are many different mRNA species that can be divided by size into three classes: 9 kB, unspliced (US) mRNA; 4 kB, singly spliced (SS) mRNA; 1.8 kB, multiply spliced (MS) mRNA. Relative levels of HIV-1 transcripts were analyzed by RTqPCR. Arrows indicating the primer binding sites of the different primer sets: US primer (purple), SS primer (green), MS primer (grey), ‘all transcripts’ primer (blue). Primer sequences see above.


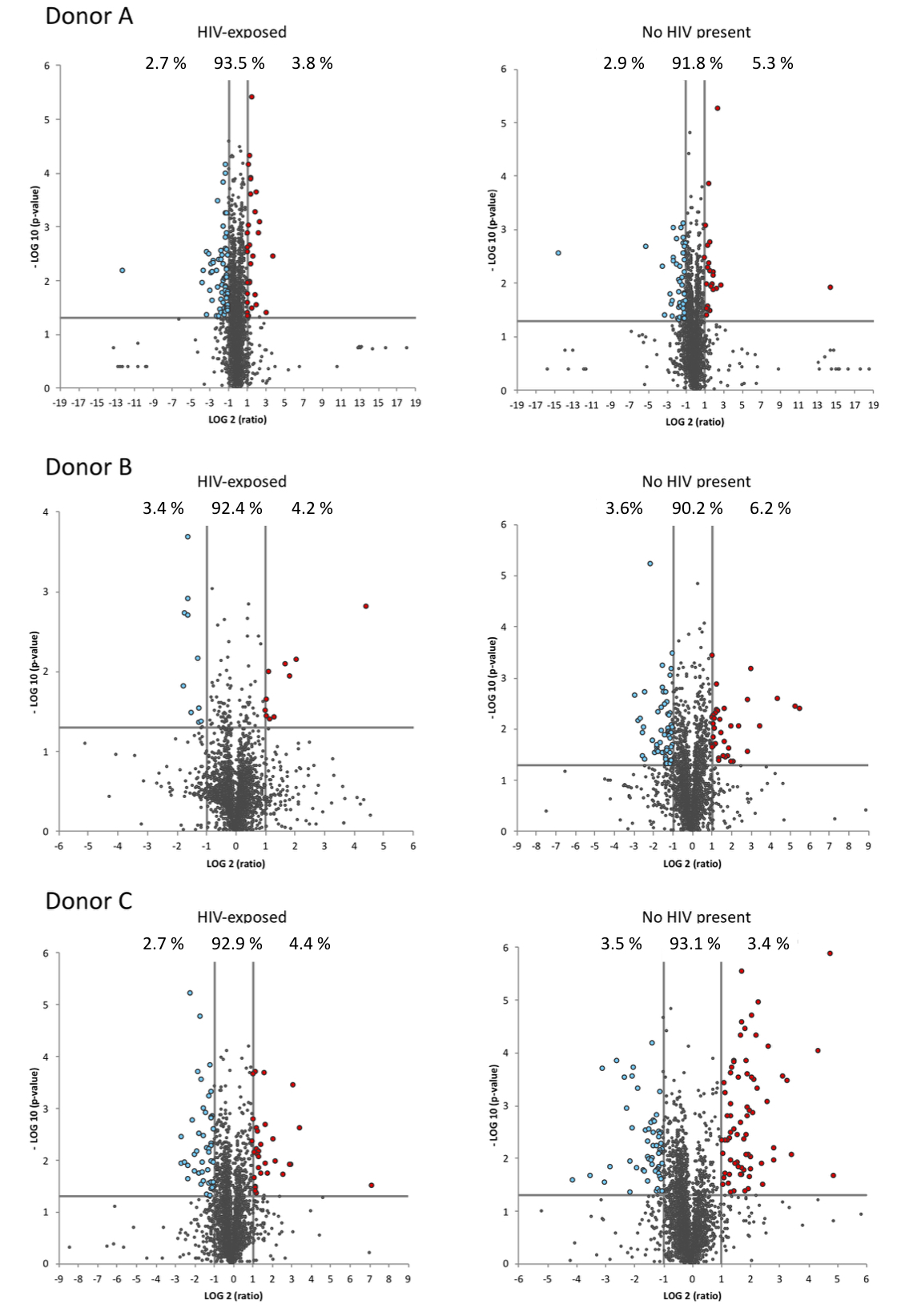


**Fig. S4. Volcano plots of proteomics analysis of PBMCs from three donors.** Compound #7 treatment affects expression levels of <10% of total proteins detected. Total percentage of proteins down-regulated (left), unchanged (centre), and up-regulated (right) in the header line. Coloured proteins showed significant changes ≥2-fold.

**
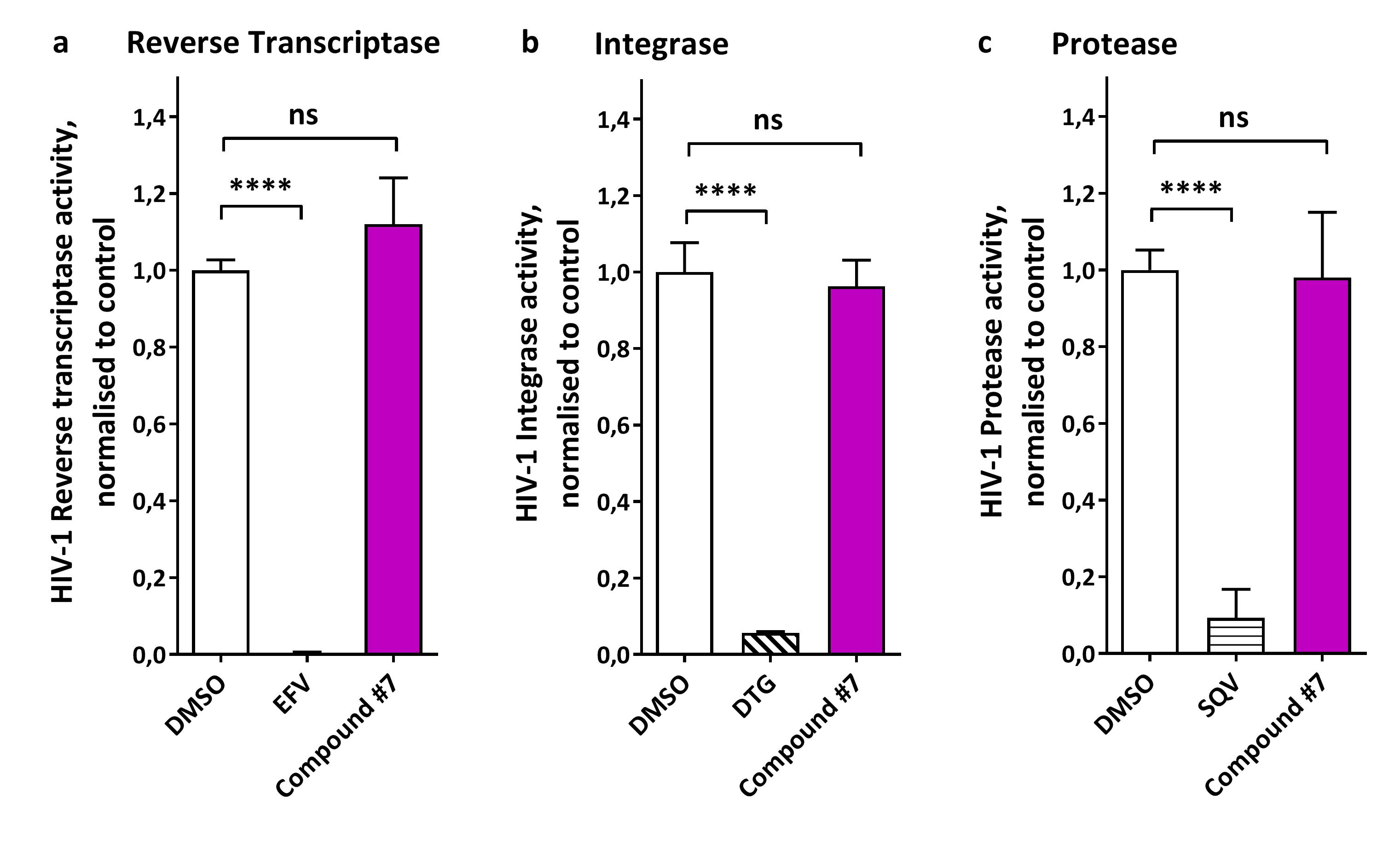
**

**Fig. S5. Biochemical assays using 10 µM compound #7 show no inhibitory effects on activities of HIV enzymes. (a)** Reverse transcriptase (reference drug: EFV; Efavirenz, 10 µM); **(b)** Integrase (reference drug: DTG; Dolutegravir, 10 µM) and **(c)** Protease (reference drug: SQV; Saquinavir, 0.1 µM). Depicted are means of three independent experiments with triplicates (n=3; m=3). Dunnett’s test was performed to determine whether treated samples were significantly different (*P*<0.05) compared to the DMSO control. *P* values are shown with asterisks indicating the level of statistical significance. *****P*≤0.0001, ns = not significant.

**Table S1.** **Anti-HIV activities of synthetic Aureothin-derivates in LC5-RIC cells.** Shown are the half maximal (IC_50_) and 90% (IC_90_) inhibitory concentrations of all Aureothin-derivatives. Data was generated by determining levels of infectious virus produced by LC5-RIC cells exposed to HIV-1_LAI_ and serial dilutions of compounds. Presented are the means of at least three independent experiments with biological triplicates (n≥3; m=3) including the standard deviation (±SD).

| # | IC_50_ (nM) | IC_90_ (nM) |
| --- | --- | --- |
| 1 | 5.3 ± 0.40 | 26.8 ± 2.54 |
| 2 | > 10,000.0 | - |
| 3 | 1.6 ± 0.40 | 579.7 ± 14.81 |
| 4 | 438.6 ± 55.11 | 3,003.0 ± 4.73 |
| 5 | 65.1 ± 6.78 | 355.2 ± 52.63 |
| 6 | 7.3 ± 0.57 | 19.9 ± 4.29 |
| 7 | 4.9 ± 0.16 | 13.4 ± 1.75 |
| 8 | 290.1 ± 53.17 | 3,525.0 ± 6.20 |
| 9 | 236.8 ± 39.71 | 1,415.0 ± 6.75 |
| 10 | 98.6 ± 10.13 | 272.6 ± 68.77 |
| 11 | 1,045.0 ± 150.20 | 8,053.0 ± 5.03 |
| 12 | 8.0 ± 1.05 | 73.7 ± 6.22 |
| 13 | 5.8 ± 0.50 | 30.1 ± 4.72 |
| 14 | 12.8 ± 0.97 | 54.3 ± 3.48 |
| 15 | 8.8 ± 0.86 | 69.7 ± 5.60 |
| 16 | 94.2 ± 9.97 | 612.1 ± 2.85 |
| 17 | 513.8 ± 40.91 | 2,938.0 ± 2.67 |
| 18 | 1,060.0 ± 67.78 | 5,915.0 ± 2.51 |
| 19 | 32.0 ± 2.22 | 151.2 ± 3.51 |
| 20 | 30.9 ± 3.70 | 367.1 ± 5.34 |
| 21 | 11.7 ± 1.38 | 163.8 ± 4.52 |

**Table S2. Antiviral activities and cell viabilities of Aureothin, compound #7 and Flavopiridol in LC5-RIC cells and PBMC.** Antiviral and cytotoxic effects of Aureothin, compound **#7** and Flavopiridol, an inhibitor of HIV transcription, were determined for LC5-RIC cells and in human peripheral blood-derived mononuclear cells (PBMC). Anti-HIV activities were determined as described in Materials and Methods. Cell viability was determined with CellTiter-Glo 2.0 Assay. Shown are the means of three independent experiments with biological triplicates (n=3; m=3) including the standard deviation (±SD).

| Compound | Anti-HIV in LC5-RIC; IC_50_ (nM) | | Cell viability in LC5-RIC; CC_50_ (nM) | Anti-HIV in PBMC; IC_50_ (nM) | Cell viability in PBMC; CC_50_ (nM) |
| --- | --- | --- | --- | --- | --- |
| Aureothin | 5.3 ± 0.40 | >10,000 | | 11.7 ± 1.37 | 2,271.0 ± 351.60 |
| Comp. #7 | 4.9 ± 0.16 | >10,000 | | 10.3 ± 0.65 | >10,000 |
| Flavopiridol | 10.9 ± 0.50 | 127.4 ± 0.94 | | 35.6 ± 3.46 | 120.1 ± 13.46 |

**Table S3. Enrichment analysis data of differentially expressed proteins in compound #7 treated PBMC and CD4+ T cells.** 289 genes up-, 372 genes down-regulated in PBMC. 76 genes up-, 177 genes down-regulated in CD4+ T cells. ^1^HIV exposed, up-regulated, ^2^HIV exposed, down-regulated, ^3^HIV unexposed, up-regulated, ^4^HIV unexposed, down-regulated

|  | **Term** | **Summary process** | **PBMC** | | | | **CD4+ T cells** | | | |
| --- | --- | --- | --- | --- | --- | --- | --- | --- | --- | --- |
|  |  |  | **^1^HIV, up** | **^2^HIV, down** | **^3^uninf, up** | **^4^uninf, down** | **^1^HIV, up** | **^2^HIV, down** | **^3^uninf, up** | **^4^uninf, down** |
| **A** | **# of GO-biological processes** | | **350** | **178** | **454** | **347** | **369** | **230** | **59** | **99** |
| **Rank 1-10**  **(p-value)** | Immune response | Immune system | 4.59E-09 |  |  | 8.45E-08 |  |  |  |  |
|  | Immune system process |  | 1.20E-08 | 5.51E-07 |  |  |  |  |  |  |
|  | Cell activation |  | 1.71E-07 |  |  |  |  |  |  |  |
| **Rank >10 (p-value)** | Viral process | Viral infection |  | 1.02E-04 | 4.24E-04 | 9.48E-05 | 6.94E-05 | 1.78E-03 |  |  |
|  | Viral life cycle |  |  | 1.24E-03 |  | 6.79E-05 | 3.03E-03 | 5.77E-04 |  |  |
|  | Response to virus |  | 9.57E-03 | 6.49E-03 | 4.55E-03 |  |  |  |  |  |
|  | Intracellular transport of virus |  |  | 8.86E-04 |  |  |  |  |  |  |
|  | Viral gene expression |  |  | 4.71E-06 |  | 2.09E-03 |  |  |  |  |
|  | Viral transcription |  |  | 8.96E-06 |  | 1.09E-03 |  |  |  |  |
|  | Immune response | Immune system |  |  | 5.72E-03 |  | 2.48E-04 |  |  | 1.14E-03 |
|  | Immune system process |  |  |  | 9.66E-14 | 6.43E-07 | 2.21E-04 |  |  | 2.61Ê-03 |
|  | Cell activation |  |  |  | 4.37E-14 | 9.65E-05 | 3.09E-03 |  | 3.10E-03 |  |
|  | Response to interferon-gamma |  |  | 7.83E-05 |  | 5.24E-04 |  |  |  |  |
|  | Leukocyte activation |  | 4.91E-05 | 3.74E-04 | 3.89E-08 | 1.11E-04 |  |  |  | 2.01E-03 |
|  | Myeloid leukocyte activation |  | 5.89E-05 |  | 3.89E-08 | 9.08E-05 | 3.09E-03 |  | 6.58E-03 | 7.50E-06 |
|  | Leukocyte migration |  | 5.19E-07 |  | 4.09E-09 |  |  |  |  |  |
|  | Leukocyte mediated immunity |  | 1.01E-05 |  | 3.13E-08 | 8.54E-04 | 3.35E-03 |  |  | 1.25E-04 |
|  | Regulation of IκB kinase/NFκB signalling |  |  | 2.30E-04 |  | 5.80E-05 |  |  |  |  |
|  | **Term** | **Summary process** | **PBMC** | | | | **CD4+ T cells** | | | |
|  |  |  | **^1^HIV, up** | **^2^HIV, down** | **^3^uninf, up** | **^4^uninf, down** | **^1^HIV, up** | **^2^HIV, down** | **^3^uninf, up** | **^4^uninf, down** |
| **B** | **# of canonical pathways** | | **26** | **8** | **23** | **15** | **46** | **9** | **1** | **7** |
| **Rank 1-10**  **(p-value)** | Haemostasis | HIV infection related | 1.72E-06 |  | 3.07E-15 |  | 6.84E-05 |  |  |  |
|  | Blood coagulation |  |  |  | 4.10E-11 |  |  |  |  |  |
|  | HIF-1α transcription factor network |  | 3.89E-05 |  | 7.36E-06 |  |  |  |  |  |
|  | Innate immune system |  | 9.53E-05 |  | 4.44E-07 |  |  |  |  | 2.48E-03 |
|  | Extracellular matrix organization |  | 1.94E-04 |  | 2.52E-06 |  |  |  |  |  |
|  | Immune system |  | 1.40E-03 |  | 6.53E-06 | 9.21E-04 |  |  |  | 6.92E-03 |
|  | HIV-1 Nef: negative effector of Fas/TNFα |  | 4.33E-04 |  |  |  |  |  |  |  |
|  | Caspase cascade in apoptosis |  | 7.35E-04 |  |  |  |  |  |  |  |
| **Rank >10 (p-value)** | Apoptotic signalling in response to DNA damage | Apoptosis | 2.15E-03 |  |  |  |  |  |  |  |
|  | Induction of apoptosis through dr3 and dr4/5 death receptors |  | 9.21E-03 |  |  |  |  |  |  |  |
|  | Apoptosis signaling pathway |  | 5.65E-03 |  |  |  | 9.48E-03 |  |  |  |
|  | FAS signalling pathway |  | 5.78E-03 |  |  |  |  |  |  |  |
|  | TNFα |  | 6.28E-03 |  |  |  | 8E+04 |  |  |  |
|  | Innate immune system | HIV infection related |  |  |  | 7.43E-03 | 2.98E-03 |  |  |  |
|  | Regulation of cell cycle progression by pIk3 |  | 4.55E-03 |  | 5.30E-03 |  |  |  |  |  |
|  | Blood coagulation |  | 9.72E-03 |  |  |  | 2.17E-03 |  |  |  |
|  | Syndecan-4-mediated signalling events |  | 3.08E-03 |  |  |  |  |  |  |  |
|  | Huntington disease |  | 7.15E-03 |  |  |  |  |  |  |  |
| **C** | **# of diseases – MesH terms** | | **2528** | **253** | **2591** | **925** | **1600** | **131** | **76** | **408** |
| **Rank 1-10**  **(p-value)** | Lymphatic diseases | Immune disease | 3.72E-16 |  |  |  |  |  |  |  |
|  | Lymphoproliferative disorders |  | 6.36E-16 |  |  |  |  |  |  |  |
|  | Immunoproliferative disorders |  | 1.56E-14 |  |  |  |  |  |  |  |
|  | Immunologic deficiency syndromes |  | 3.56E-15 |  |  |  |  |  |  |  |
|  | **Term** | **Summary process** | **PBMC** | | | | **CD4+ T cells** | | | |
|  |  |  | **^1^HIV, up** | **^2^HIV, down** | **^3^uninf, up** | **^4^uninf, down** | **^1^HIV, up** | **^2^HIV, down** | **^3^uninf, up** | **^4^uninf, down** |
|  | RNA virus infections | Immune disease | 4.91E-15 |  |  |  |  |  |  |  |
|  | Lentivirus infections |  | 2.86E-14 |  |  |  |  |  |  |  |
| **Rank >10 (p-value)** | Sexually Transmitted Diseases | Immune disease |  |  |  |  | 3.17E-03 |  |  | 3.25E-04 |
|  | Lymphatic diseases |  |  |  | 2.43E-15 |  | 3.96E-03 | 1.49E-03 |  |  |
|  | Lymphoproliferative disorders |  |  |  | 8.28E-13 | 7.96E-04 | 7.8E-04 |  |  |  |
|  | Immunoproliferative disorders |  |  |  | 8.28E-13 | 5.66E-04 | 7.53E-04 | 9.09E-03 |  |  |
|  | Immunologic deficiency syndromes |  |  | 8.14E-05 | 7.17E-15 | 5.80E-07 | 3.00E-03 | 1.44E-03 |  | 8.06E-04 |
|  | Virus Diseases |  |  |  |  |  | 4.51E-05 | 8.76E-03 |  | 2.49E-04 |
|  | Lentivirus infections |  |  | 1.36E-04 | 1.99E-16 | 7.99E-07 | 9.73E-04 | 9.79E-03 |  | 1.78E-03 |
|  | RNA virus infections |  |  | 1.43E-04 | 1.92E-17 | 1.63E-05 | 1.14E-05 | 6.45E-03 |  | 1.37E-04 |
|  | HIV infections |  | 7.51E-14 | 1.53E-04 | 4.97E-16 | 1.67E-06 | 6.89E-04 |  |  | 7.70E-04 |
|  | DNA virus infections |  | 3.18E-08 | 1.81E-04 | 2.85E-13 | 1.01E-05 | 1.08E-03 |  |  | 3.25E-04 |
|  | Metabolic Diseases |  |  |  |  |  | 3.47E-05 |  |  |  |
|  | Retroviridae infections |  | 4.23E-13 | 8.33E-05 | 5.32E-16 | 9.52E-07 | 2.89E-03 | 8.99E-03 |  | 4.12E-03 |
|  | Mitochondrial Diseases |  |  |  |  |  | 1.87E-03 | 3.12E-03 |  |  |
| **D** | **# of unigene tissue terms** | | **79** | **82** | **80** | **81** | **76** | **76** | **2** | **48** |
| **Rank 1-10**  **(p-value)** | Haemolymphoid system | Blood cells | 6.49E-17 | 5.15E-30 | 1.76E-12 | 1.03E-34 | 9.79E-26 | 9.82E-38 |  | 1.27E-10 |
|  | Blood |  |  | 1.93E-26 |  | 2.23E-37 |  | 9.82E-38 |  |  |
|  | Leukocyte |  |  |  |  | 5.31E-29 |  | 1.75E-29 |  |  |
| **Rank >10 (p-value)** | Blood | Blood cells | 2.28E-11 |  | 1.36E-09 |  | 6.54E-19 |  |  | 7.53E-06 |
|  | Leukocyte |  |  | 3.54E-20 | 5.17E-09 |  | 7.10E-18 |  |  | 6.16E-06 |

**Supplementary data file S1. ^1^H-NMR spectra of Aureothin-derivatives.**

**Supplementary data file S2. Proteins detected by proteomics analysis of PBMC & CD4+ T cells.**

**Supplementary data file S3. Detailed enrichment analysis of PBMC & CD4+ T cell proteomics results.**

1 Balachandran, A. & Cochrane, A. Screening for small molecule inhibitors of HIV-1 Gag expression. *Methods* **126**, 201-208, doi:10.1016/j.ymeth.2017.06.007 (2017).

2 Schneider, M. *et al.* A new model for post-integration latency in macroglial cells to study HIV-1 reservoirs of the brain. *AIDS* **29**, 1147-1159, doi:10.1097/QAD.0000000000000691 (2015).

3 Radonic, A. *et al.* Guideline to reference gene selection for quantitative real-time PCR. *Biochem Biophys Res Commun* **313**, 856-862 (2004).

4 Grosche, A. *et al.* The Proteome of Native Adult Muller Glial Cells From Murine Retina. *Mol Cell Proteomics* **15**, 462-480, doi:10.1074/mcp.M115.052183 (2016).

5 Wisniewski, J. R., Zougman, A., Nagaraj, N. & Mann, M. Universal sample preparation method for proteome analysis. *Nat Methods* **6**, 359-362, doi:10.1038/nmeth.1322 (2009).

6 Lepper, M. F. *et al.* Proteomic Landscape of Patient-Derived CD4+ T Cells in Recent-Onset Type 1 Diabetes. *J Proteome Res* **17**, 618-634, doi:10.1021/acs.jproteome.7b00712 (2018).

7 Brosch, M., Yu, L., Hubbard, T. & Choudhary, J. Accurate and sensitive peptide identification with Mascot Percolator. *J Proteome Res* **8**, 3176-3181, doi:10.1021/pr800982s (2009).
